# Supplementary material for: Molecular basis of nick ligation in the nucleosome by DNA Ligase IIIα
Source: Nat Commun. 2026 Sep 2;17:10460. doi: 10.1038/s41467-026-77207-7 (PMC13635136; doi:10.1038/s41467-026-77207-7)
Supplement: Supplementary file 1 [file 41467_2026_77207_MOESM1_ESM.pdf]

Supplementary Information File

“Molecular basis of nick ligation in the nucleosome by DNA Ligase IIIα”

Boesch and Martin et al.

Supplementary Fig. 1: Validation of recombinant NCPs and recombinant proteins

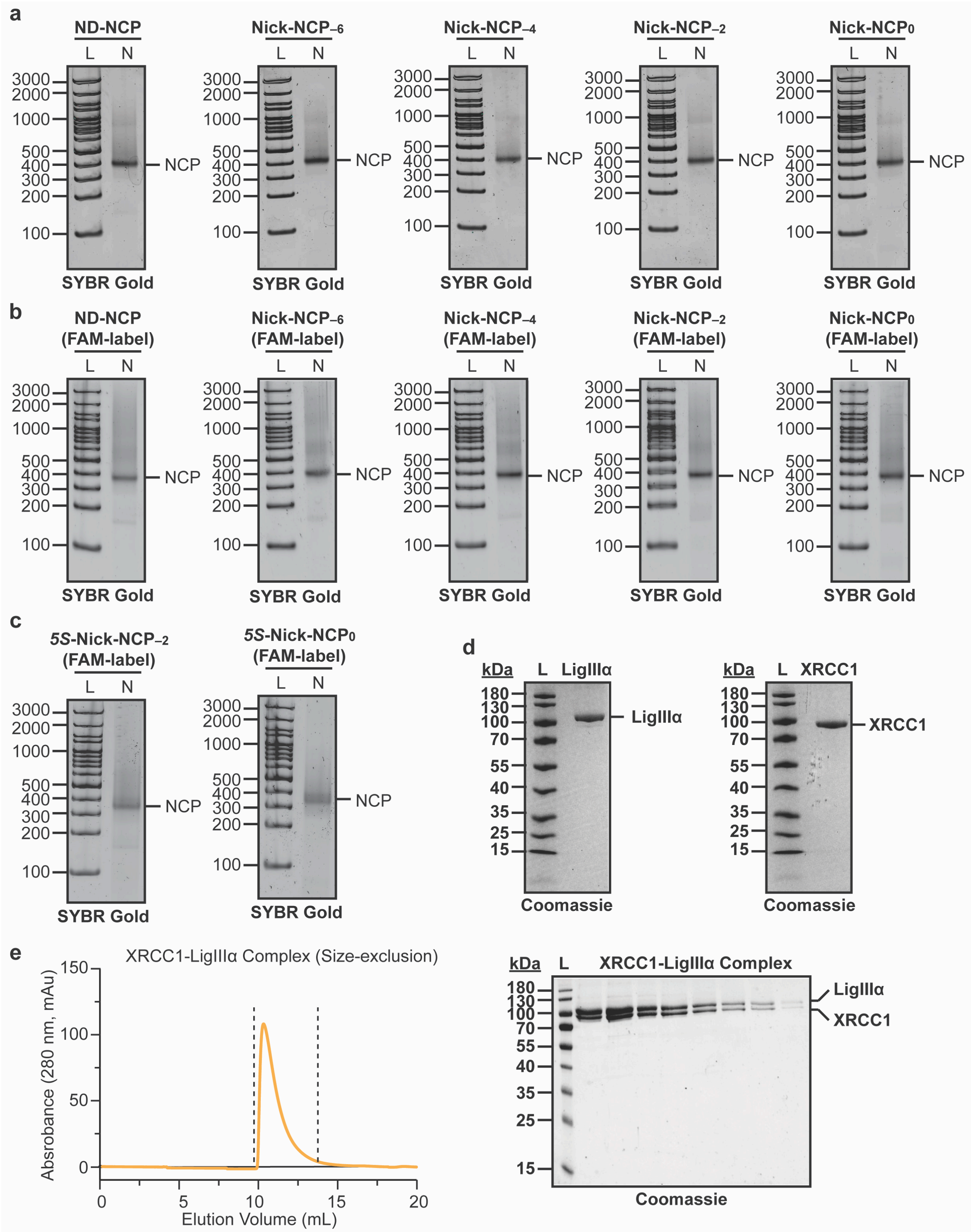

Supplementary Fig. 1: Validation of recombinant NCPs and recombinant proteins

a, Native PAGE gels confirming nucleosome formation and purity for the non-damaged NCP (ND-NCP), Nick-NCP$_{-6}$, Nick-NCP$_{-4}$, Nick-NCP$_{-2}$, and Nick-NCP$_{0}$ samples. The NCPs were detected using SYBR Gold staining. The lanes corresponding to 100 bp DNA ladder (L) and nucleosome sample (N) are labeled. **b,** Native PAGE gels confirming nucleosome formation and purity for the 6-carboxyfluorescein (FAM)-labeled ND-NCP, Nick-NCP$_{-6}$, Nick-NCP$_{-4}$, Nick-NCP$_{-2}$, and Nick-NCP$_{0}$ samples. The NCPs were detected using SYBR Gold staining. The lanes corresponding to a 100 bp DNA ladder (L) and nucleosome sample (N) are labeled. **c,** Native PAGE gels confirming nucleosome formation and purity for the FAM-labeled *5S*-Nick-NCP$_{-2}$, and *5S*-Nick-NCP$_{0}$ samples. The NCPs were detected using SYBR Gold staining. The lanes corresponding to a 100 bp DNA ladder (L) and nucleosome sample (N) are labeled. **d,** SDS-PAGE gels confirming the purity of recombinant LigIIIα (left) and recombinant XRCC1 (right). The proteins were detected with Coomassie blue staining. The lanes corresponding to the protein ladder (L) are labeled. **e,** Size-exclusion chromatogram (left) and associated SDS-PAGE gel (right) confirming the purity of the recombinant XRCC1-LigIIIα complex. The proteins were detected with Coomassie blue staining. The lanes corresponding to the protein ladder (L) are labeled. All source data in this figure are provided as a Source data file.

Supplementary Fig. 2: Single-turnover kinetic analysis of LigIIIα nick ligation in the nucleosome

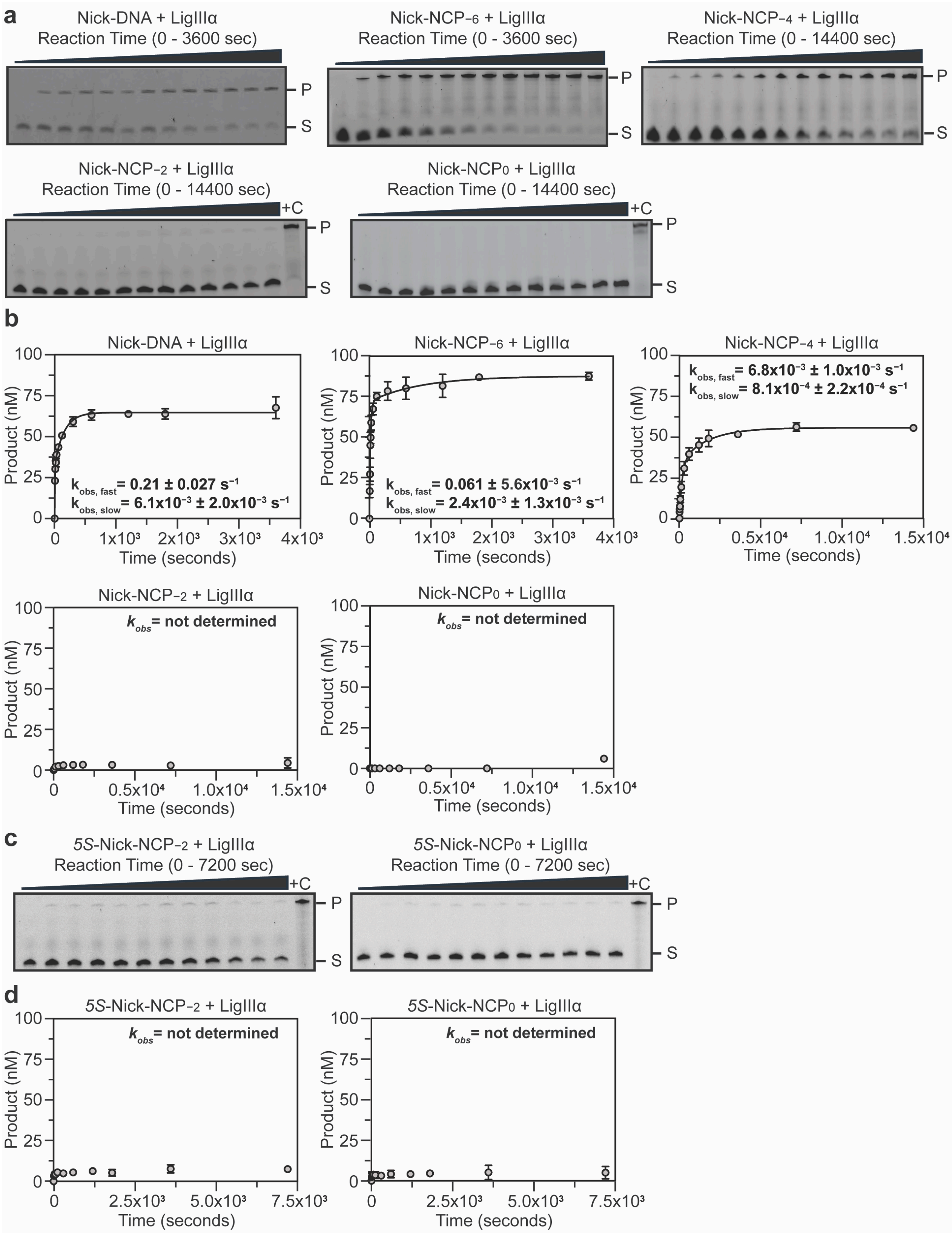

Supplementary Fig. 2: Single-turnover kinetic analysis of LigIIIα nick ligation in the nucleosome

a, Representative denaturing Urea-PAGE gels from the single-turnover kinetic experiments for LigIIIα and Nick-DNA, Nick-NCP$_{-6}$, Nick-NCP$_{-4}$, Nick-NCP$_{-2}$, and Nick-NCP$_{0}$. The substrate (S, Nick-DNA or Nick-NCP) and product (P, ligated DNA or NCP) were detected using the 6-FAM label on the nucleosomal DNA of each NCP. A 147 nt single-stranded DNA was loaded as a positive control (+C) for experiments with less than 10% product formed over the kinetic time course. The gels are representative of three independent single-turnover kinetic experiments performed for LigIIIα and the Nick-DNA and each Nick-NCP. **b,** Quantification of the single-turnover kinetic experiments for LigIIIα and Nick-DNA, Nick-NCP$_{-6}$, Nick-NCP$_{-4}$, Nick-NCP$_{-2}$, and Nick-NCP$_{0}$. The data points represent the mean ± standard deviation from three independent replicate experiments. The error bars are included for all experimental data points, but some error bars are smaller than the circles used to represent data points. The ligation rate (k_{obs}) is shown as an inset for each experiment and represents the mean ± standard error of the mean from the three independent replicate experiments. The ligation rate (k_{obs}) was not determined for experiments with less than 10% product formed over the kinetic time course. **c,** Representative denaturing Urea-PAGE gels from the single-turnover kinetic experiments for LigIIIα and *5S*-Nick-NCP$_{-2}$ and *5S*-Nick-NCP$_{0}$. The substrate (S, Nick-NCP) and product (P, ligated NCP) were detected using the 6-FAM label on the nucleosomal DNA of each NCP. A 147 nt single-stranded DNA was loaded as a positive control (+C) for experiments with less than 10% product formed over the kinetic time course. The gels are representative of three independent single-turnover kinetic experiments performed for LigIIIα and the *5S*-Nick-NCP$_{-2}$ and *5S*-Nick-NCP$_{0}$ **d,** Quantification of the single-turnover kinetic experiments for LigIIIα and *5S*-Nick-NCP$_{-2}$ and *5S*-Nick-NCP$_{0}$. The data points represent the mean ± standard deviation from three independent replicate experiments. The error bars are included for all experimental data points, but some error bars are smaller than the circles used to represent data points. The ligation rate (k_{obs}) was not determined for experiments with less than 10% product formed over the kinetic time course. All source data in this figure are provided as a Source Data file.

Supplementary Fig. 3: EMSA Analysis of the LigIIIα-Nick-NCP interaction

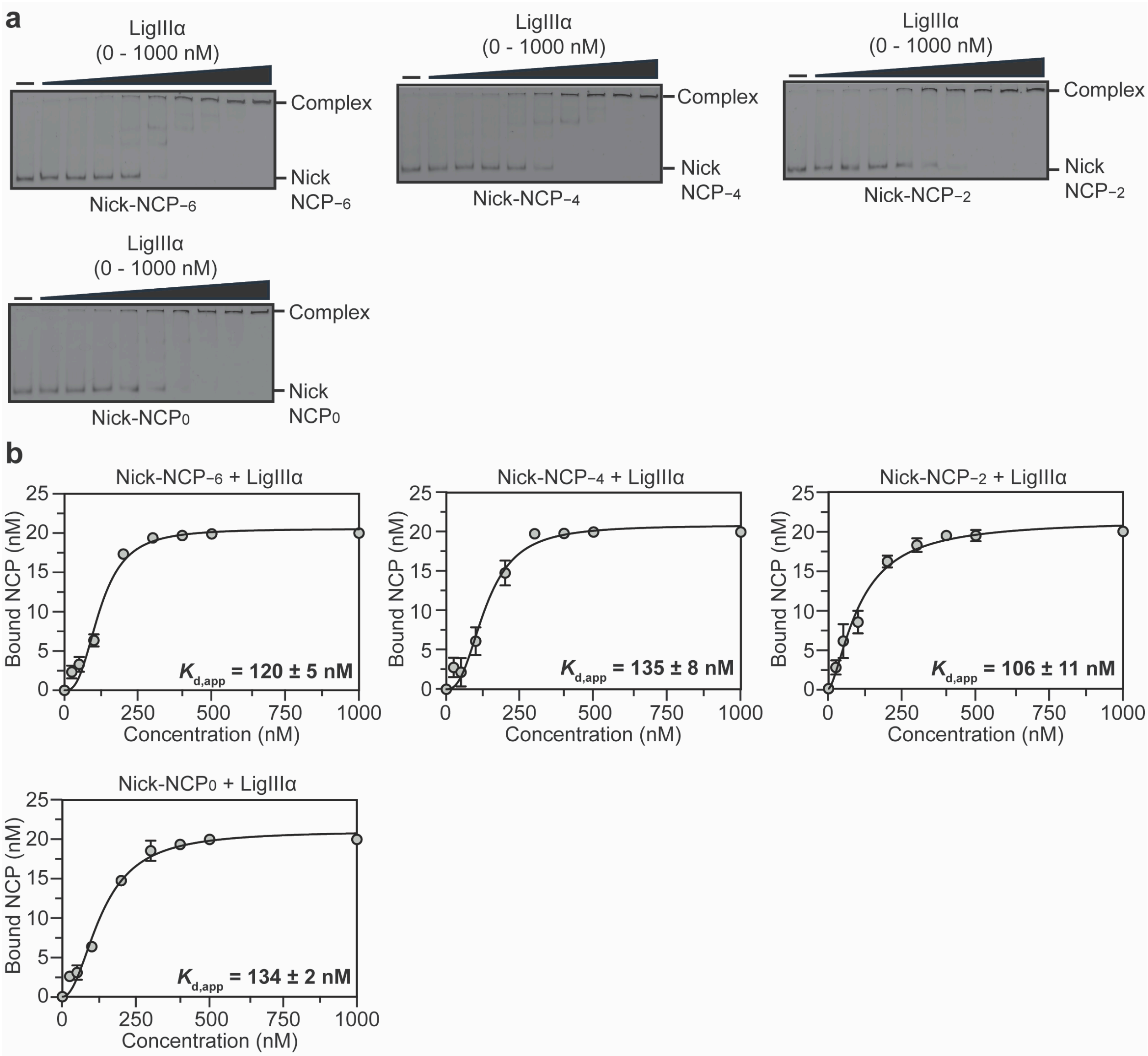

Supplementary Fig. 3: EMSA Analysis of the LigIIIα-Nick-NCP interaction

a, Representative native PAGE gels from the electrophoretic mobility shift assays (EMSAs) for LigIIIα and Nick-NCP$_{-6}$, Nick-NCP$_{-4}$, Nick-NCP$_{-2}$, and Nick-NCP$_{0}$. The free Nick-NCP and LigIIIα-Nick-NCP complex were detected using the 6-FAM label on the nucleosomal DNA of each NCP. The gels are representative of three independent EMSA experiments performed for LigIIIα and each Nick-NCP. **b,** Quantification of the EMSA experiments for LigIIIα and Nick-NCP$_{-6}$, Nick-NCP$_{-4}$, Nick-NCP$_{-2}$, and Nick-NCP$_{0}$. The data points represent the mean ± standard deviation from the three independent replicate experiments. The error bars are included for all experimental data points, but some error bars are smaller than the circles used to represent data points. The apparent binding affinity ($K_{d,app}$) is shown as an inset for each experiment and represents the mean ± standard deviation from the three independent replicate experiments. All source data in this figure are provided as a Source Data file.

Supplementary Fig. 4: ND-NCP single particle analysis processing workflow

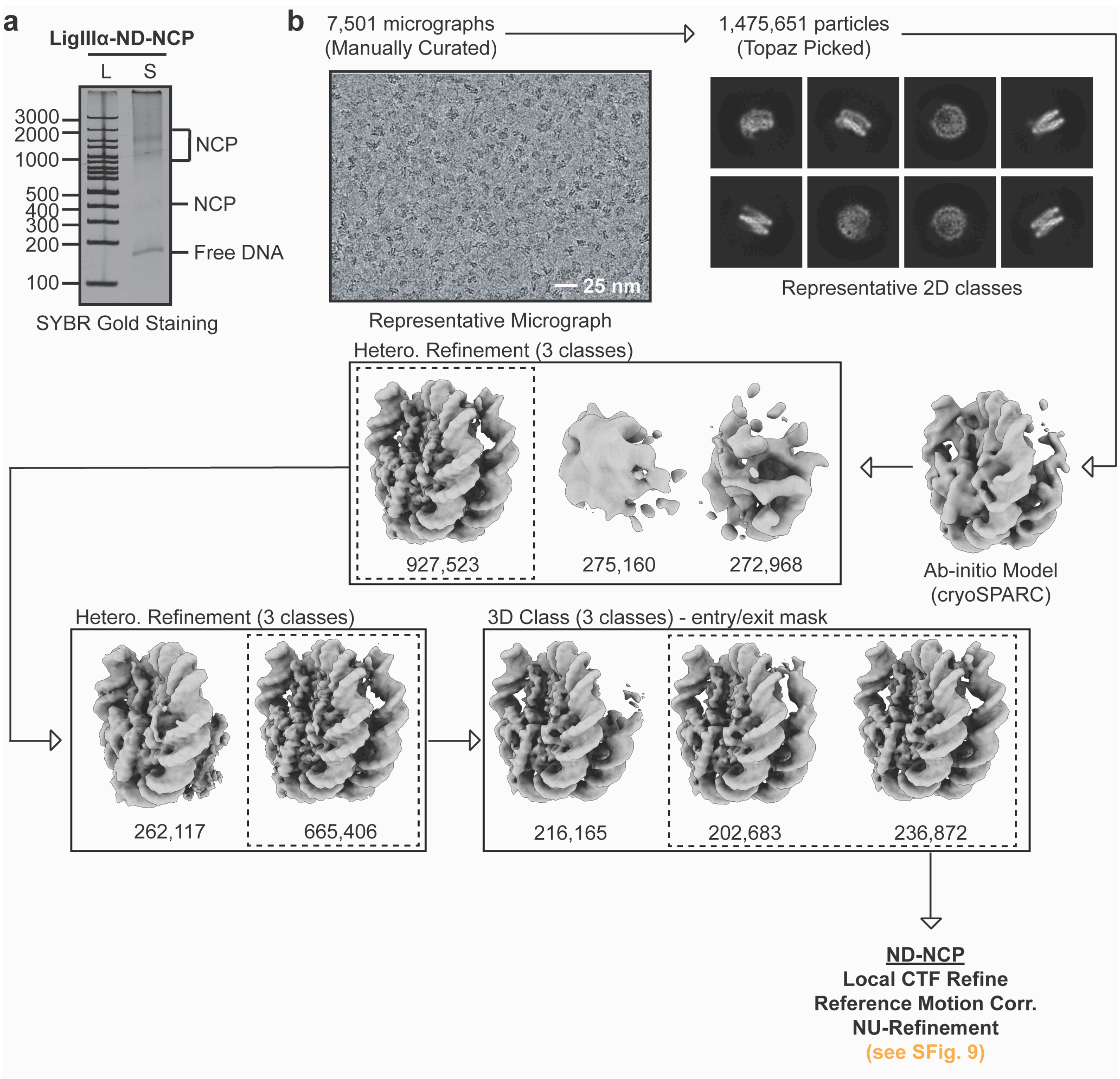

Supplementary Fig. 4: ND-NCP single particle analysis processing workflow

a, Native PAGE gel of the ND-NCP cryo-EM sample (S) and a 100 bp DNA ladder (L). The ND-NCP complexes were visualized with SYBR gold staining. **b,** Cryo-EM data processing workflow for the ND-NCP dataset. A representative micrograph (n=7,501) and representative 2D classes from the ND-NCP dataset are shown. The maps chosen for further classification and refinement throughout the cryo-EM data processing workflow are boxed. The final map, final model, and quality assessment metrics for ND-NCP can be found in Supplementary Fig. 9. All source data in this figure are provided as a Source data file.

Supplementary Fig. 5: LigIIIα-Nick-NCP-6 single particle analysis processing workflow

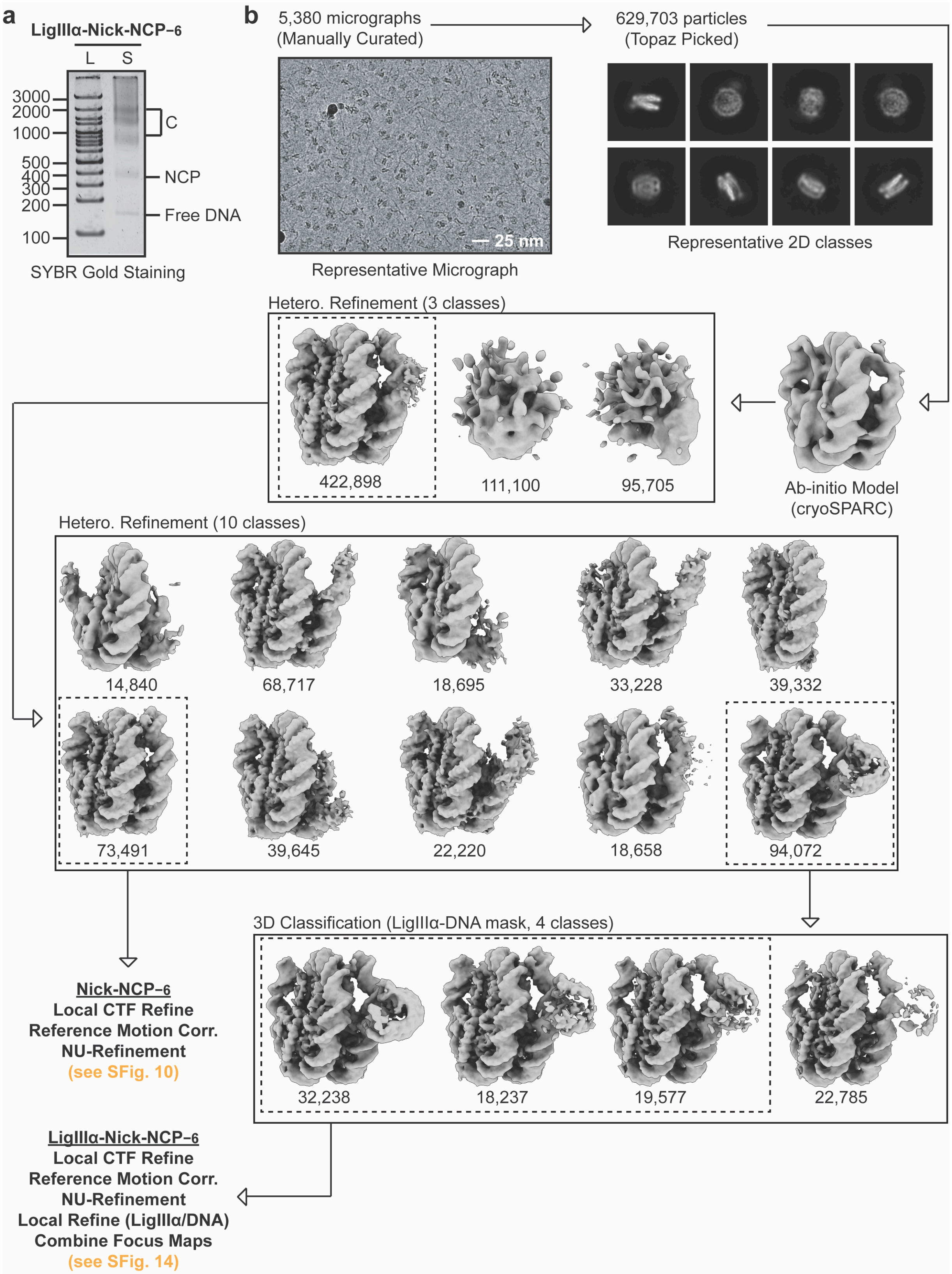

Supplementary Fig. 5: LigIIIα-Nick-NCP$_{-6}$ single particle analysis processing workflow

a, Native PAGE gel of the LigIIIα-Nick-NCP$_{-6}$ cryo-EM sample (S) and a 100 bp DNA ladder (L). The NCP$_{-6}$ and LigIIIα-Nick-NCP$_{-6}$ complex were visualized with SYBR gold staining. The bands corresponding to free DNA, the NCP, and LigIIIα-Nick-NCP$_{-6}$ (C) are labeled. **b,** Cryo-EM data processing workflow for the LigIIIα-Nick-NCP$_{-6}$ dataset. A representative micrograph (n=5,380) and representative 2D classes from the LigIIIα-Nick-NCP$_{-6}$ dataset are shown. The maps chosen for further classification and/or refinement throughout the data processing pipeline are boxed. The final maps, final models, and quality assessment metrics for Nick-NCP$_{-6}$ and LigIIIα-Nick-NCP$_{-6}$ can be found in Supplementary Fig. 10 and Supplementary Fig. 14, respectively. All source data in this figure are provided as a Source data file.

Supplementary Fig. 6: LigIIIα-Nick-NCP-4 single particle analysis processing workflow

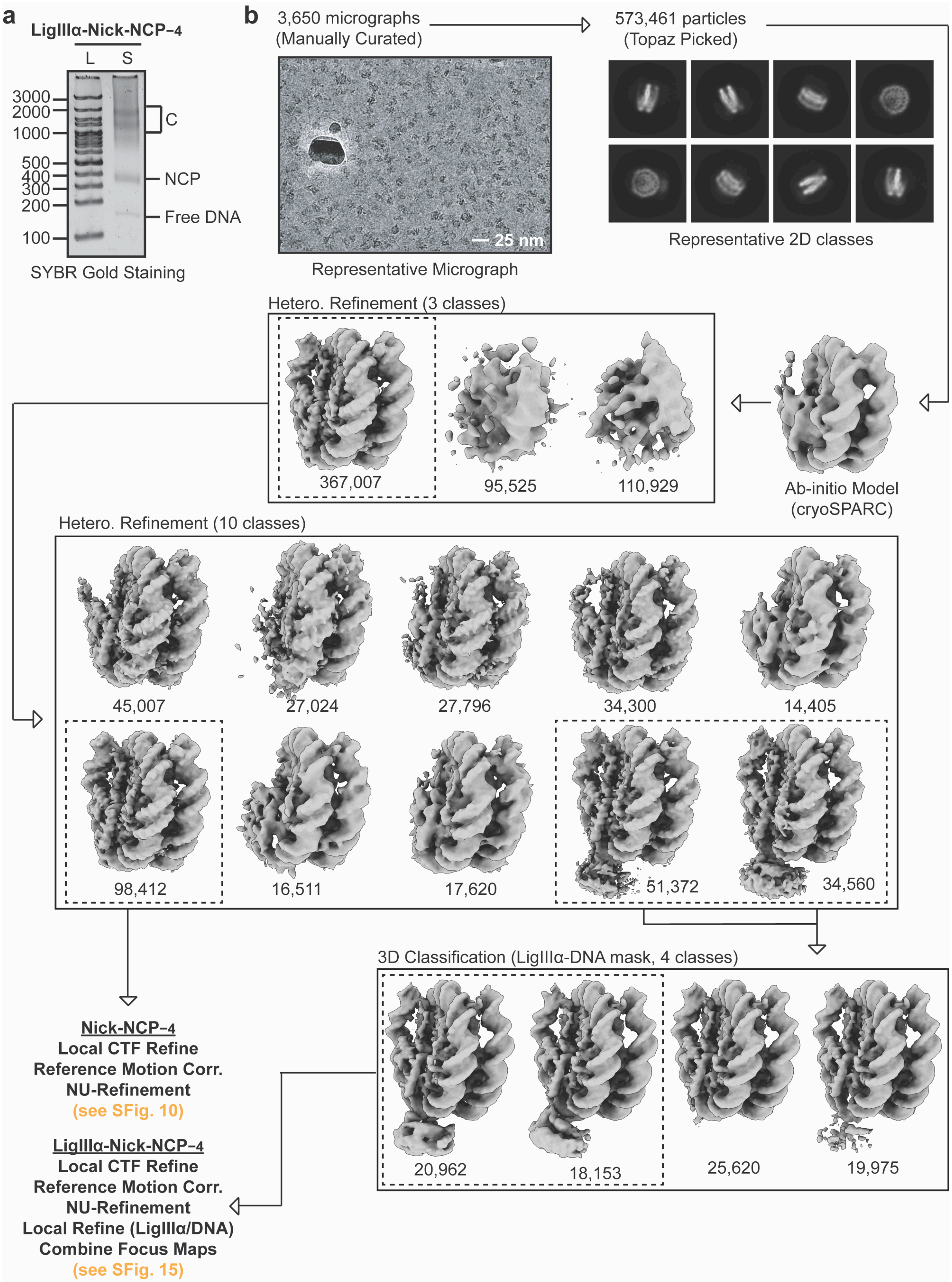

Supplementary Fig. 6: LigIIIα-Nick-NCP$_{-4}$ single particle analysis processing workflow

a, Native PAGE gel of the LigIIIα-Nick-NCP$_{-4}$ cryo-EM sample (S) and a 100 bp DNA ladder (L). The NCP$_{-4}$ and LigIIIα-Nick-NCP$_{-4}$ complex were visualized with SYBR gold staining. The bands corresponding to free DNA, the NCP, and LigIIIα-Nick-NCP$_{-4}$ (C) are labeled. **b,** Cryo-EM data processing workflow for the LigIIIα-Nick-NCP$_{-4}$ dataset. A representative micrograph (n=3,650) and representative 2D classes from the LigIIIα-Nick-NCP$_{-4}$ dataset are shown. The maps chosen for further classification and/or refinement throughout the data processing pipeline are boxed. The final maps, final models, and quality assessment metrics for Nick-NCP$_{-4}$ and LigIIIα-Nick-NCP$_{-4}$ can be found in Supplementary Fig. 10 and Supplementary Fig. 15, respectively. All source data in this figure are provided as a Source data file.

Supplementary Fig. 7: LigIIIα-Nick-NCP–2 single particle analysis processing workflow

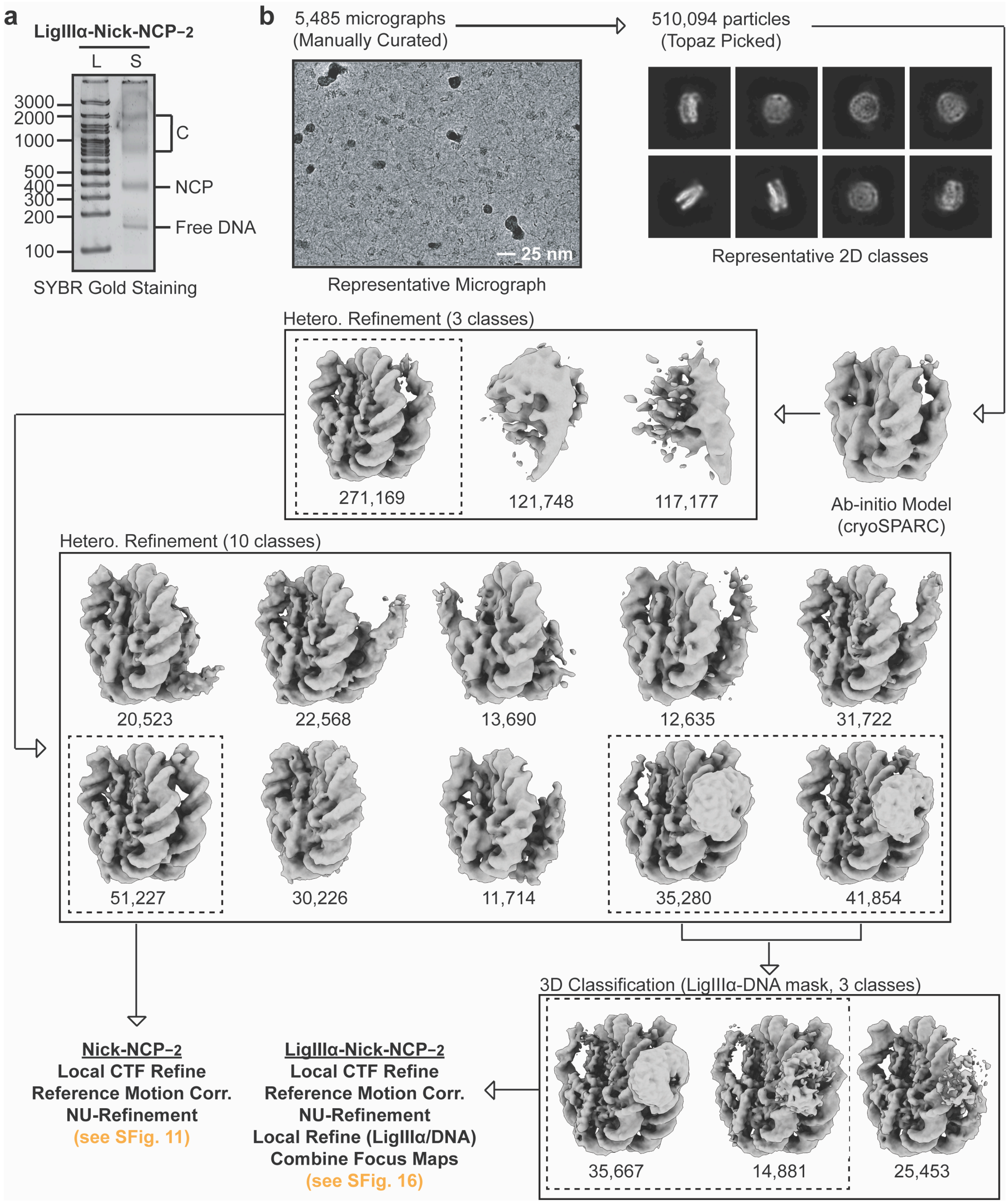

Supplementary Fig. 7: LigIIIα-Nick-NCP$_{-2}$ single particle analysis processing workflow

a, Native PAGE gel of the LigIIIα-Nick-NCP$_{-2}$ cryo-EM sample (S) and a 100 bp DNA ladder (L). The NCP$_{-2}$ and LigIIIα-Nick-NCP$_{-2}$ complex were visualized with SYBR gold staining. The bands corresponding to free DNA, the NCP, and LigIIIα-Nick-NCP$_{-2}$ (C) are labeled. **b,** Cryo-EM data processing workflow for the LigIIIα-Nick-NCP$_{-2}$ dataset. A representative micrograph (n=5,485) and representative 2D classes from the LigIIIα-Nick-NCP$_{-2}$ dataset are shown. The maps chosen for further classification and/or refinement throughout the data processing pipeline are boxed. The final maps, final models, and quality assessment metrics for Nick-NCP$_{-2}$ and LigIIIα-Nick-NCP$_{-2}$ can be found in Supplementary Fig. 11 and Supplementary Fig. 16, respectively. All source data in this figure are provided as a Source data file.

Supplementary Fig. 8: LigIIIα-Nick-NCP0 single particle analysis processing workflow

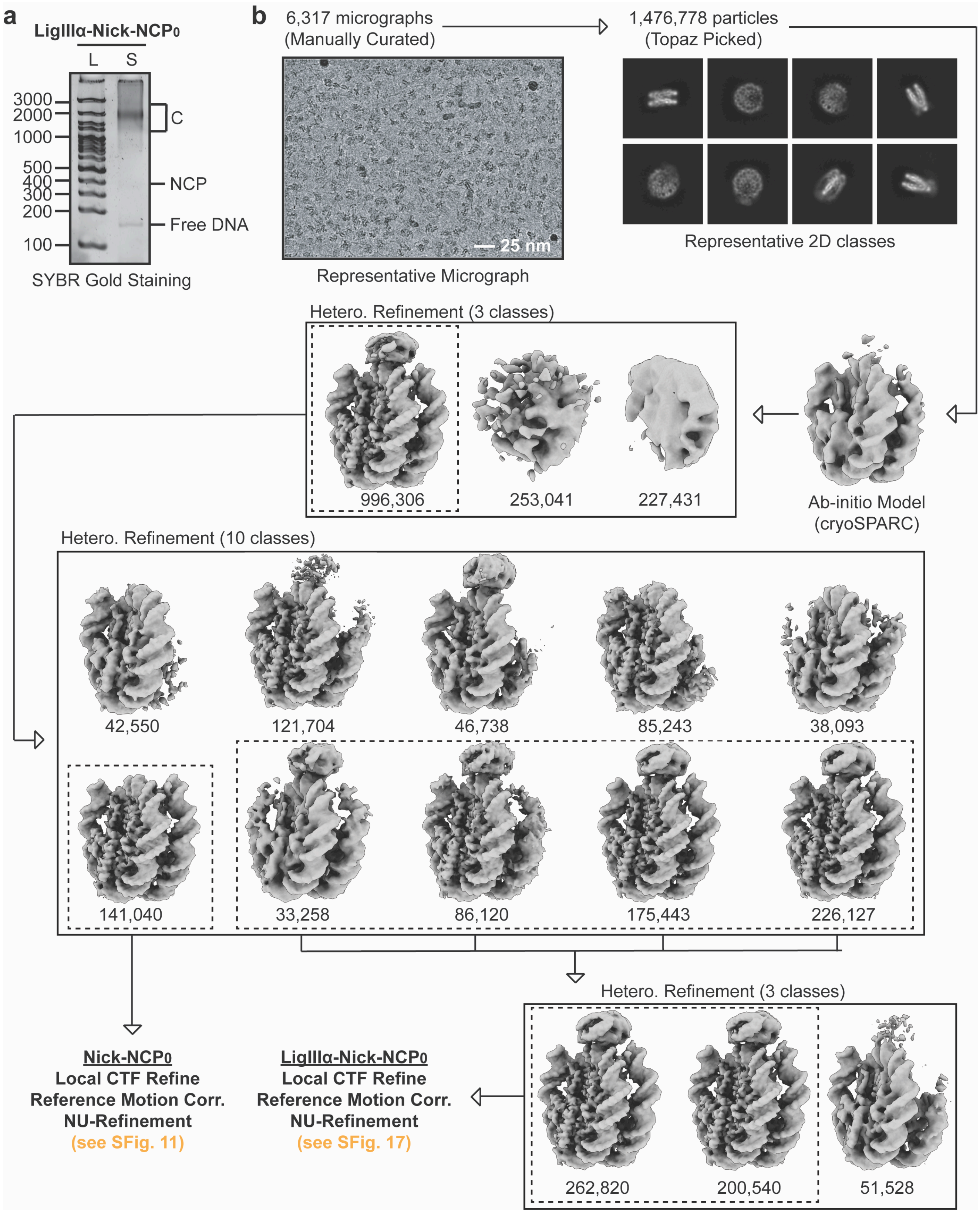

Supplementary Fig. 8: LigIIIα-Nick-NCP0 single particle analysis processing workflow

a, Native PAGE gel of the LigIIIα-Nick-NCP0 cryo-EM sample (S) and a 100 bp DNA ladder (L). The NCP0 and LigIIIα-Nick-NCP0 complex were visualized with SYBR gold staining. The bands corresponding to free DNA, the NCP, and LigIIIα-Nick-NCP0 (C) are labeled. **b,** Cryo-EM data processing workflow for the LigIIIα-Nick-NCP0 dataset. A representative micrograph (n=6,317) and representative 2D classes from the LigIIIα-Nick-NCP0 dataset are shown. The maps chosen for further classification and/or refinement throughout the data processing pipeline are boxed. The final maps, final models, and quality assessment metrics for Nick-NCP0 and LigIIIα-Nick-NCP0 can be found in Supplementary Fig. 11 and Supplementary Fig. 17, respectively. All source data in this figure are provided as a Source data file.

Supplementary Fig. 9: ND-NCP map and model quality assessment

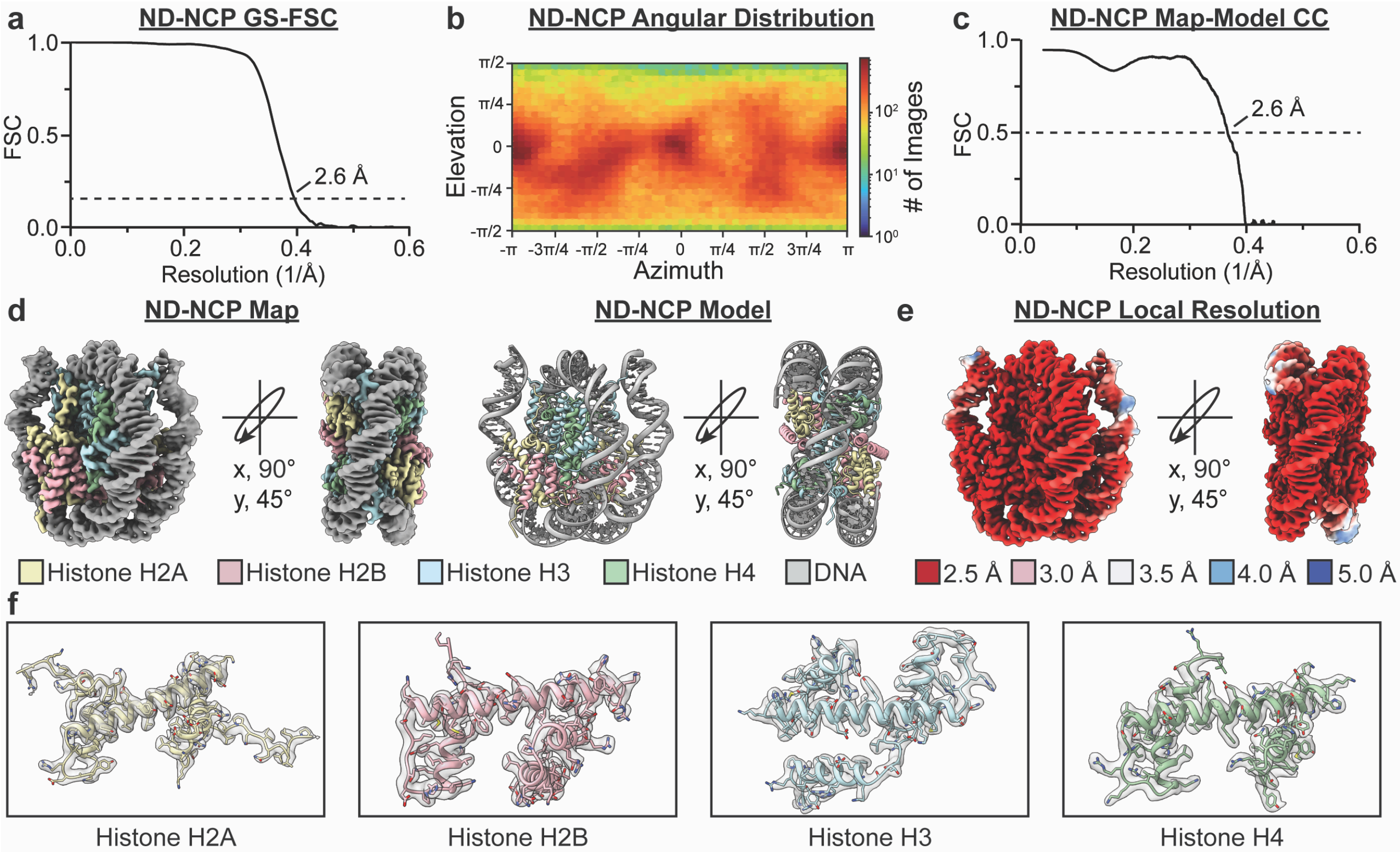

Supplementary Fig. 9: ND-NCP map and model quality assessment

a, Gold-standard Fourier shell correlation (GS-FSC) for the ND-NCP cryo-EM map (solid black line). The dashed line corresponds to GS-FSC - 0.143. **b,** Angular distribution heatmap for the ND-NCP cryo-EM map**. c,** Map-to-model FSC for the ND-NCP model and cryo-EM map. The dashed line corresponds to FSC - 0.5. **d,** The final 2.6 Å ND-NCP cryo-EM map and model shown in two different orientations. **e**, Local resolution estimation for the ND-NCP cryo-EM map shown in two different orientations. **f,** Representative segmented densities for histones H2A, H2B, H3, and H4 from the ND-NCP cryo-EM map. The representative segmented densities from the cryo-EM map are shown as transparent gray surfaces. All source data in this figure are provided as a Source data file.

Supplementary Fig. 10: Nick-NCP−6 and Nick-NCP−4 map and model quality assessment

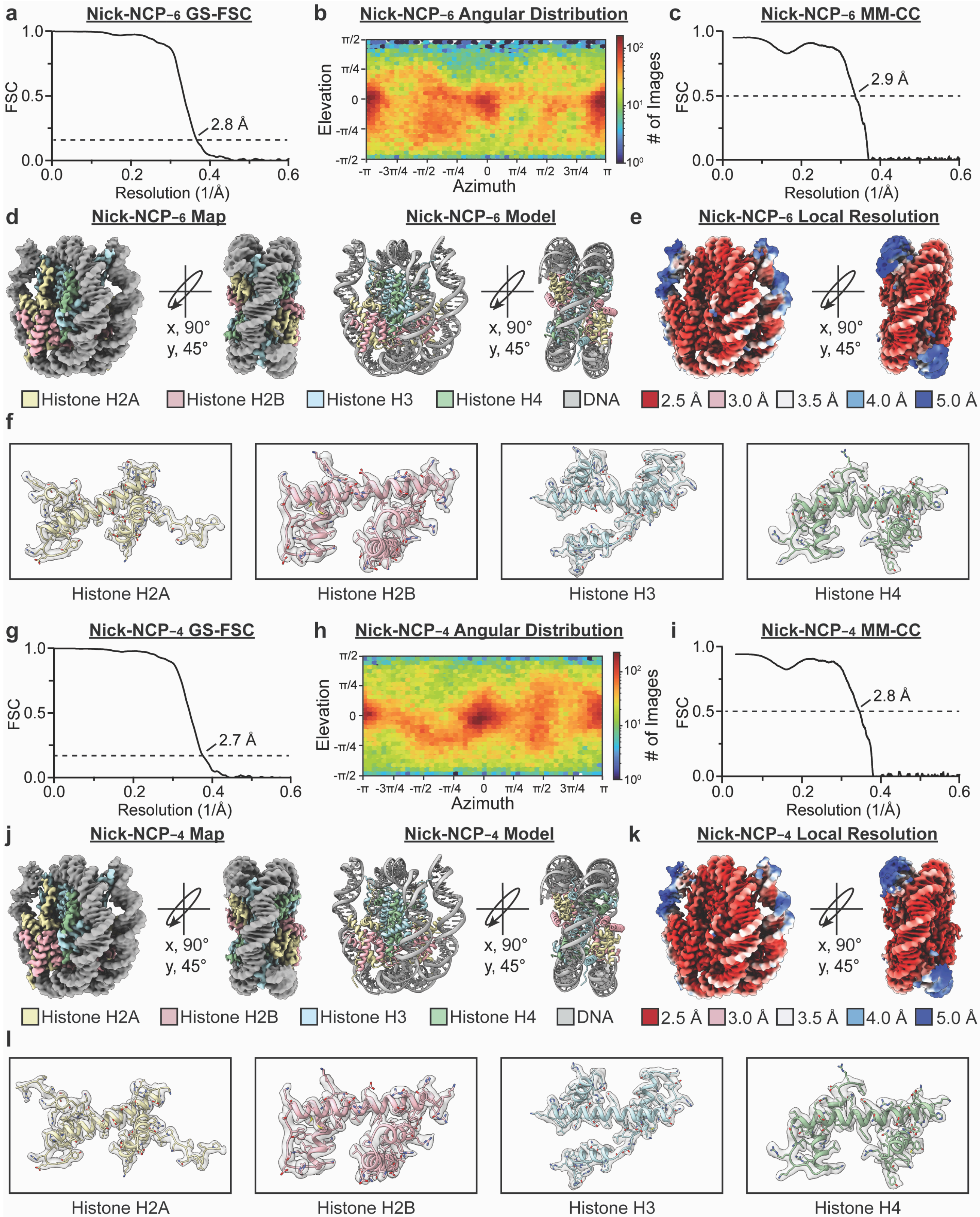

Supplementary Fig. 10: Nick-NCP$_{-6}$ and Nick-NCP$_{-4}$ map and model quality assessment

a, Gold-standard Fourier shell correlation (GS-FSC) for the Nick-NCP$_{-6}$ cryo-EM map (solid black line). The dashed line corresponds to GS-FSC - 0.143. **b,** Angular distribution heatmap for the Nick-NCP$_{-6}$ cryo-EM map**.** **c,** Map-to-model FSC for the Nick-NCP$_{-6}$ model and cryo-EM map. The dashed line corresponds to FSC - 0.5. **d,** The final 2.8 Å Nick-NCP$_{-6}$ cryo-EM map and model shown in two different orientations. **e**, Local resolution estimation for the Nick-NCP$_{-6}$ cryo-EM map shown in two different orientations. **f,** Representative segmented densities for histones H2A, H2B, H3, and H4 from the Nick-NCP$_{-6}$ cryo-EM map. The representative segmented densities from the cryo-EM map are shown as transparent gray surfaces. **g,** Gold-standard Fourier shell correlation (GS-FSC) for the Nick-NCP$_{-4}$ cryo-EM map (solid black line). The dashed line corresponds to GS-FSC - 0.143. **h,** Angular distribution heatmap for the Nick-NCP$_{-4}$ cryo-EM map**.** **i,** Map-to-model FSC for the Nick-NCP$_{-4}$ model and cryo-EM map. The dashed line corresponds to FSC - 0.5. **j,** The final 2.7 Å Nick-NCP$_{-4}$ cryo-EM map and model shown in two different orientations. **k**, Local resolution estimation for the Nick-NCP$_{-4}$ cryo-EM map shown in two different orientations. **l,** Representative segmented densities for histones H2A, H2B, H3, and H4 from the Nick-NCP$_{-4}$ cryo-EM map. The representative segmented densities from the cryo-EM map are shown as transparent gray surfaces. All source data in this figure are provided as a Source data file.

Supplementary Fig. 11: Nick-NCP−2 and Nick-NCP0 map and model quality assessment

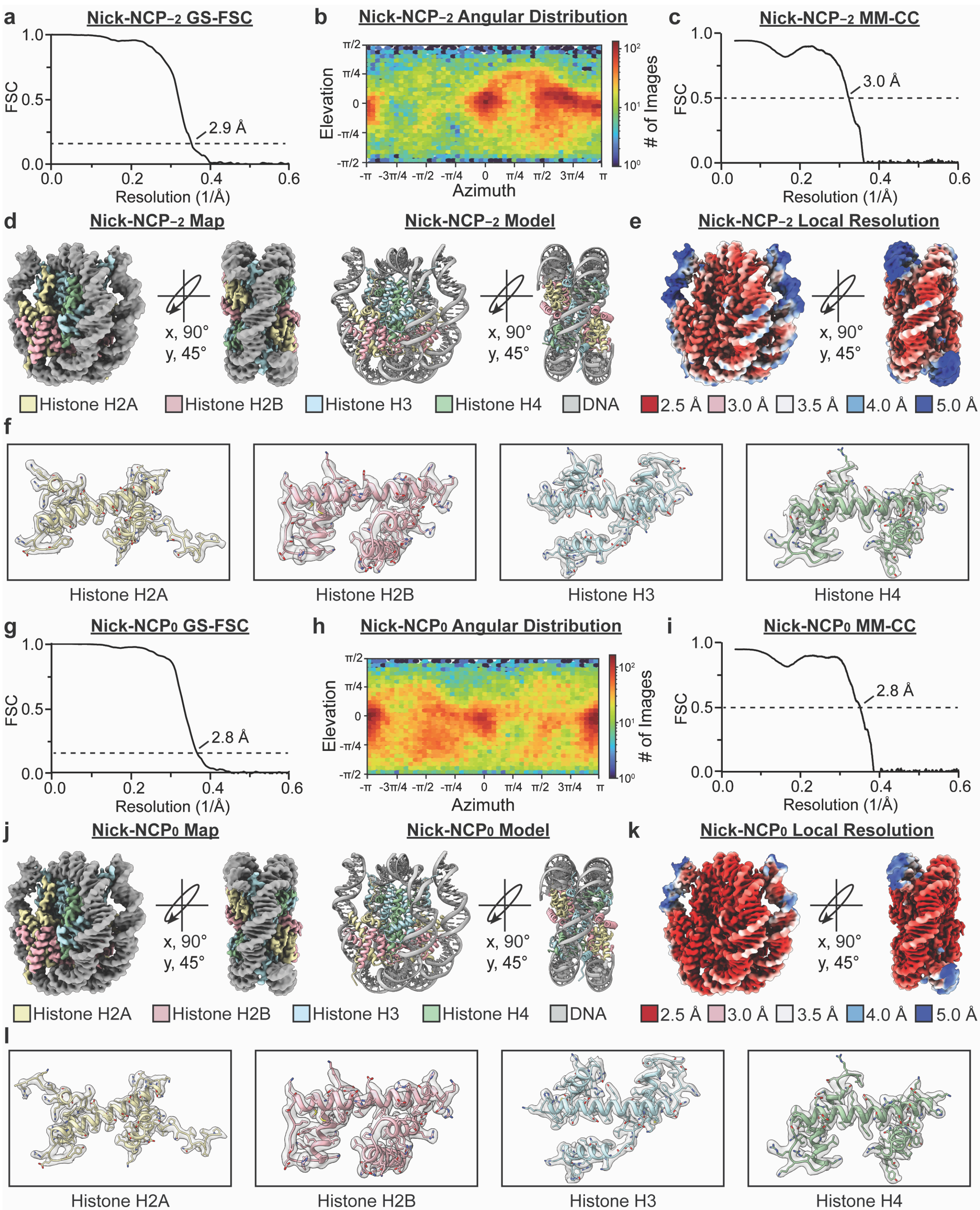

Supplementary Fig. 11: Nick-NCP$_{-2}$ and Nick-NCP$_0$ map and model quality assessment

a, Gold-standard Fourier shell correlation (GS-FSC) for the Nick-NCP$_{-2}$ cryo-EM map (solid black line). The dashed line corresponds to GS-FSC - 0.143. **b,** Angular distribution heatmap for the Nick-NCP$_{-2}$ cryo-EM map**.** **c,** Map-to-model FSC for the Nick-NCP$_{-2}$ model and cryo-EM map. The dashed line corresponds to FSC - 0.5. **d,** The final 2.9 Å Nick-NCP$_{-2}$ cryo-EM map and model shown in two different orientations. **e**, The local resolution estimation for the Nick-NCP$_{-2}$ cryo-EM map shown in two different orientations. **f,** Representative segmented densities for histones H2A, H2B, H3, and H4 from the Nick-NCP$_{-2}$ cryo-EM map. The representative segmented densities from the cryo-EM map are shown as transparent gray surfaces. **g,** Gold-standard Fourier shell correlation (GS-FSC) for the Nick-NCP$_0$ cryo-EM map (solid black line). The dashed line corresponds to GS-FSC - 0.143. **h,** Angular distribution heatmap for the Nick-NCP$_0$ cryo-EM map**.** **i,** Map-to-model FSC for the Nick-NCP$_0$ model and cryo-EM map. The dashed line corresponds to FSC - 0.5. **j,** The final 2.8 Å Nick-NCP$_0$ cryo-EM map and model shown in two different orientations. **k**, Local resolution estimation for the Nick-NCP$_0$ cryo-EM map shown in two different orientations. **l,** Representative segmented densities for histones H2A, H2B, H3, and H4 from the Nick-NCP$_0$ cryo-EM map. The representative segmented densities from the cryo-EM map are shown as transparent gray surfaces. All source data in this figure are provided as a Source data file.

Supplementary Fig. 12: Nicks have minimal impact on nucleosome stability and structure

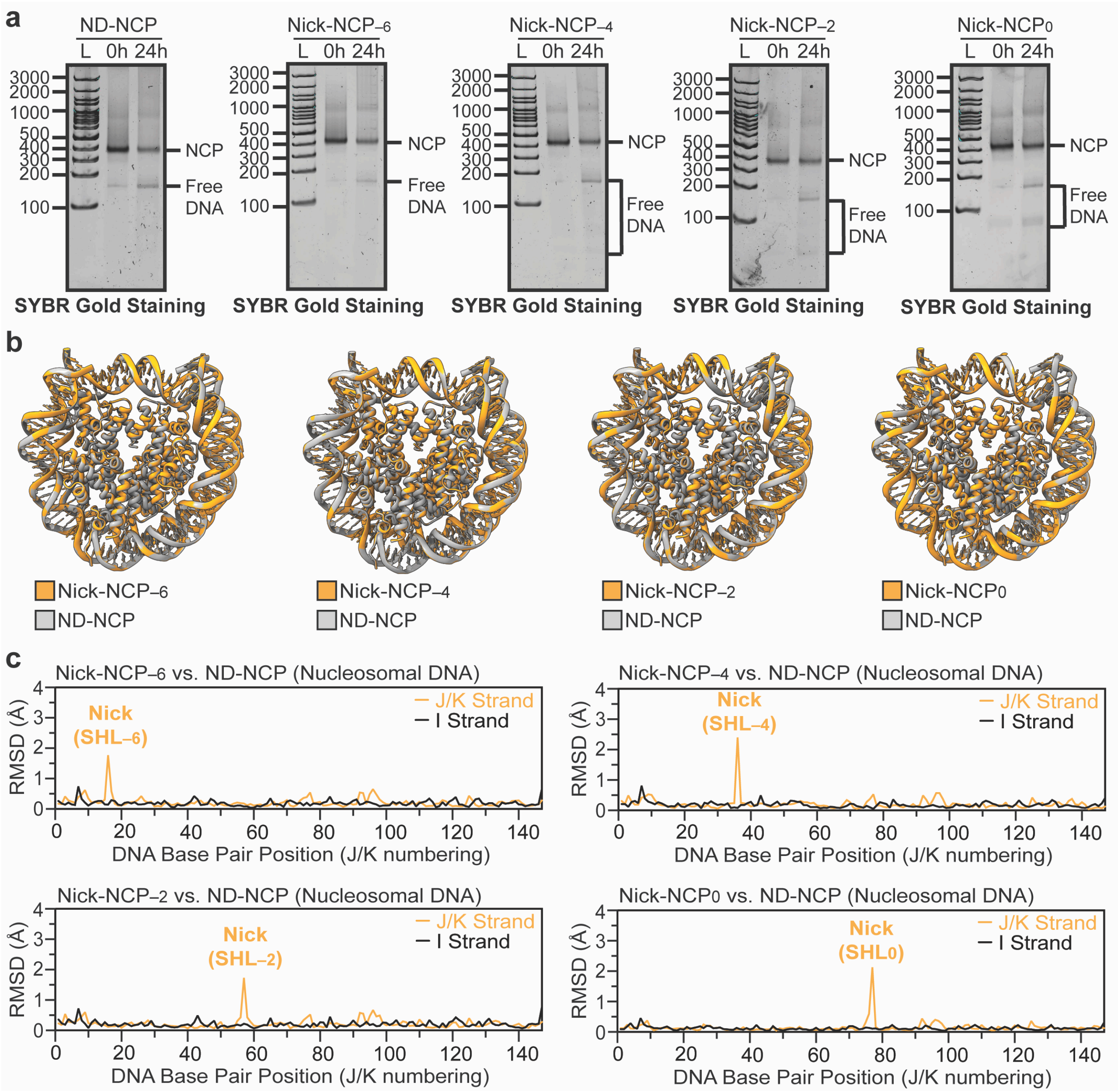

Supplementary Fig. 12: Nicks have minimal impact on nucleosome stability and structure

a, Representative native PAGE gels from the Nick-NCP$_{-6}$, Nick-NCP$_{-4}$, Nick-NCP$_{-2}$, and Nick-NCP$_{0}$ nucleosome stability assays. The NCP and free DNA were detected using SYBR Gold staining. The gels are representative of three independent nucleosome stability assays performed for each Nick-NCP. **b,** Structural comparison of the ND-NCP with Nick-NCP$_{-6}$ (left), Nick-NCP$_{-4}$ (left middle), Nick-NCP$_{-2}$ (right middle), and Nick-NCP$_{0}$ (right). **c,** Root mean square deviation (RMSD) analysis comparing the nucleosomal DNA in the ND-NCP with the nucleosomal DNA in Nick-NCP$_{-6}$ (left), Nick-NCP$_{-4}$ (left middle), Nick-NCP$_{-2}$ (right middle), and Nick-NCP$_{0}$ (right). The location of the nick within the nucleosomal DNA is labeled in orange. All source data in this figure are provided as a Source data file.

Supplementary Fig. 13: Nicks have minimal impact on fast timescale nucleosome dynamics

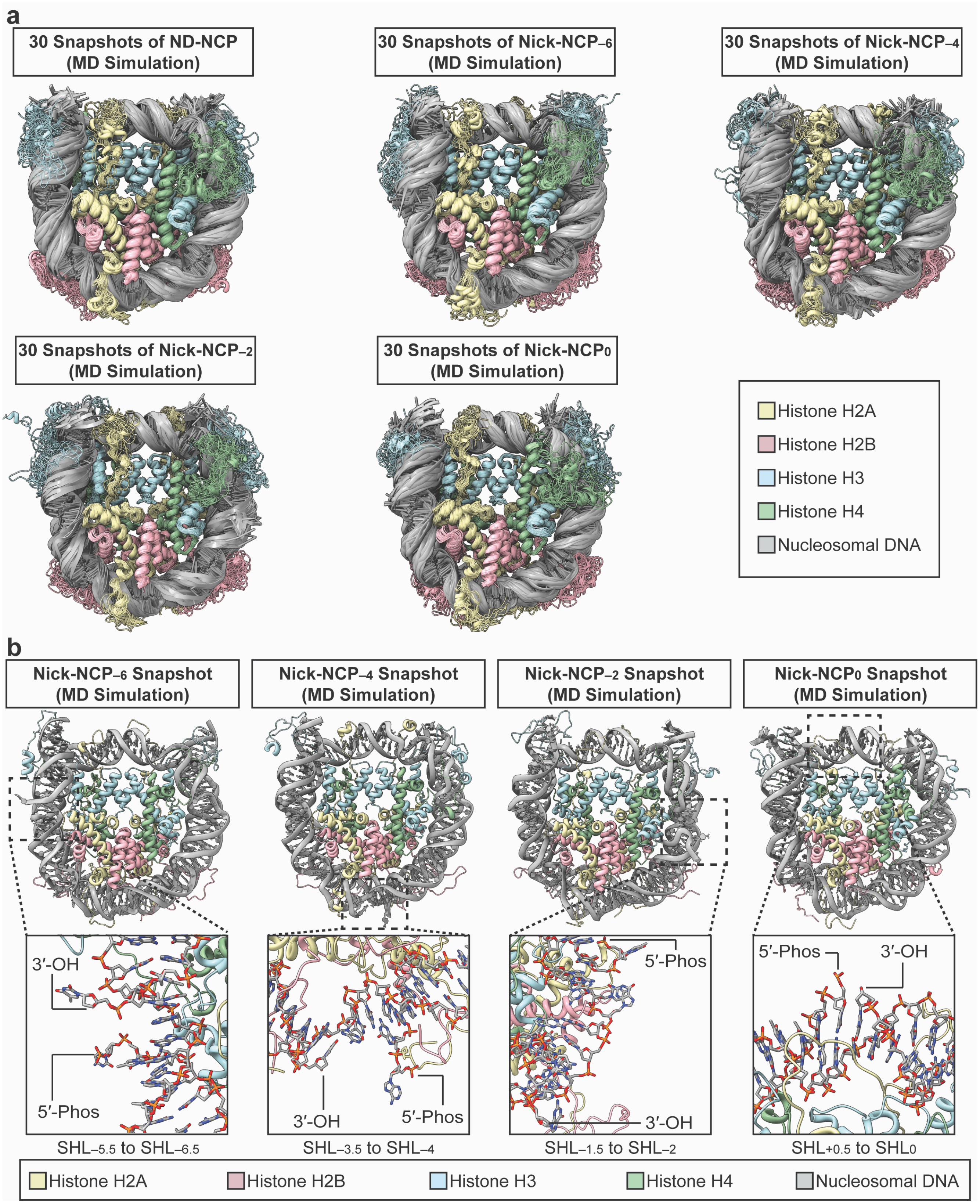

Supplementary Fig. 13: Nicks have minimal impact on fast timescale nucleosome dynamics

a, Overlay of 30 structural snapshots equally spaced throughout the 1 μs MD simulation for ND-NCP (top left), Nick-NCP$_{-6}$ (top middle), Nick-NCP$_{-4}$ (top right), Nick-NCP$_{-2}$ (bottom left), and Nick-NCP$_{0}$ (bottom right). **b,** A structural snapshot of Nick-NCP$_{-6}$ (left), Nick-NCP$_{-4}$ (middle left), Nick-NCP$_{-2}$ (middle right), and Nick-NCP$_{0}$ (right) from the 1 μs MD simulations with a focused view of the nick site shown as an inset.

Supplementary Fig. 14: LigIIIα-Nick-NCP−6 map and model quality assessment

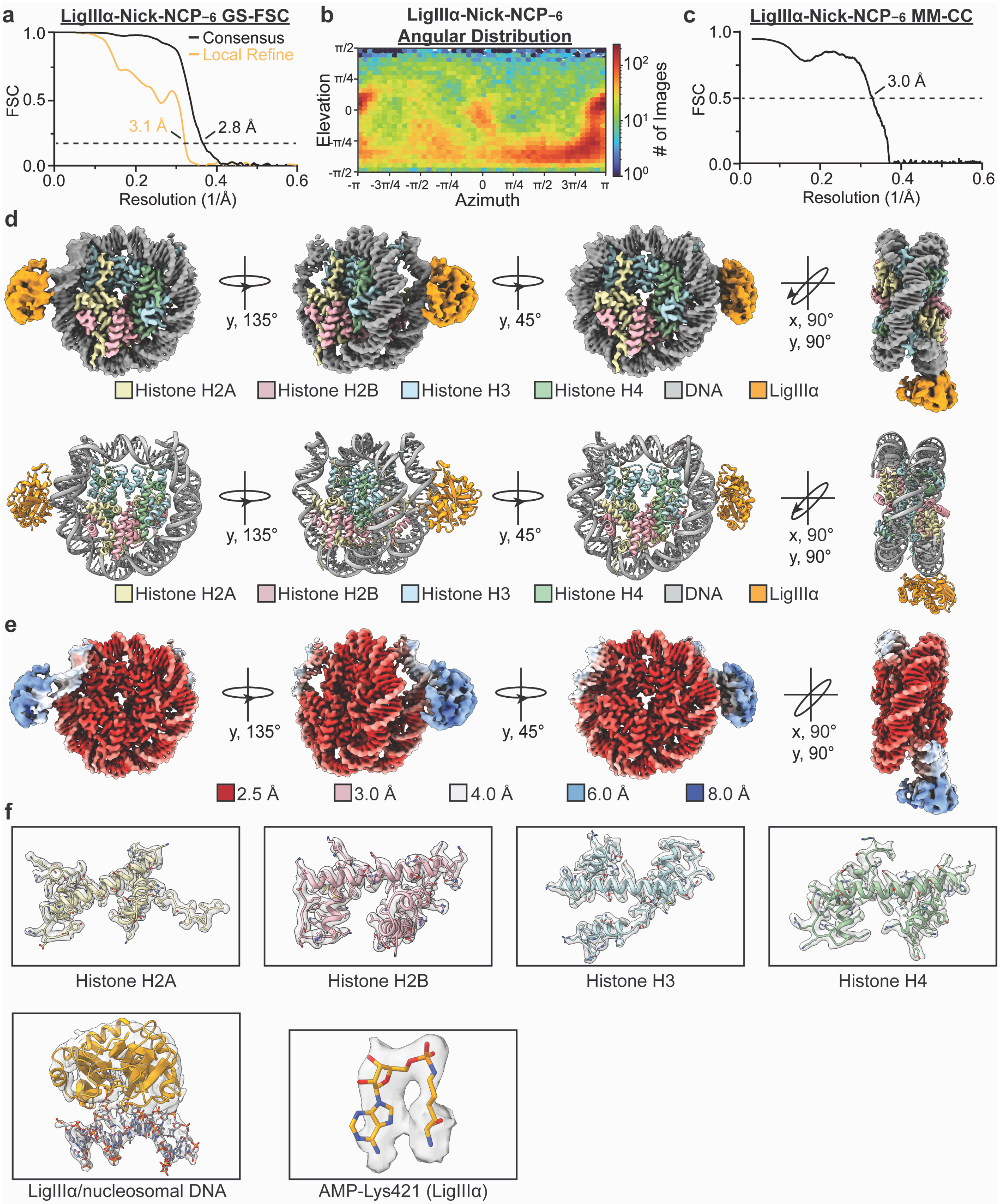

Supplementary Fig. 14: LigIIIα-Nick-NCP$_{-6}$ map and model quality assessment

a, Gold-standard Fourier shell correlation (GS-FSC) for the LigIIIα-Nick-NCP$_{-6}$ consensus cryo-EM map (solid black line) and the LigIIIα/nucleosomal DNA focus cryo-EM map (solid orange line). The dashed line corresponds to GS-FSC - 0.143. **b,** Angular distribution heatmap for the LigIIIα-Nick-NCP$_{-6}$ composite cryo-EM map. **c,** Map-to-model FSC for the LigIIIα-Nick-NCP$_{-6}$ model and composite cryo-EM map. The dashed line corresponds to FSC - 0.5. **d,** The final 2.8 Å LigIIIα-Nick-NCP$_{-6}$ composite cryo-EM map and model shown in four different orientations. **e**, Local resolution estimation for the LigIIIα-Nick-NCP$_{-6}$ composite cryo-EM map shown in four different orientations. **f,** Representative segmented densities for histones H2A, H2B, H3, H4, the LigIIIα/nucleosomal DNA binding interface, and the adenylylated-K421 from the LigIIIα-Nick-NCP$_{-6}$ composite cryo-EM map. The representative segmented densities from the cryo-EM map are shown as transparent gray surfaces. All source data in this figure are provided as a Source data file.

Supplementary Fig. 15: LigIIIα-Nick-NCP−4 map and model quality assessment

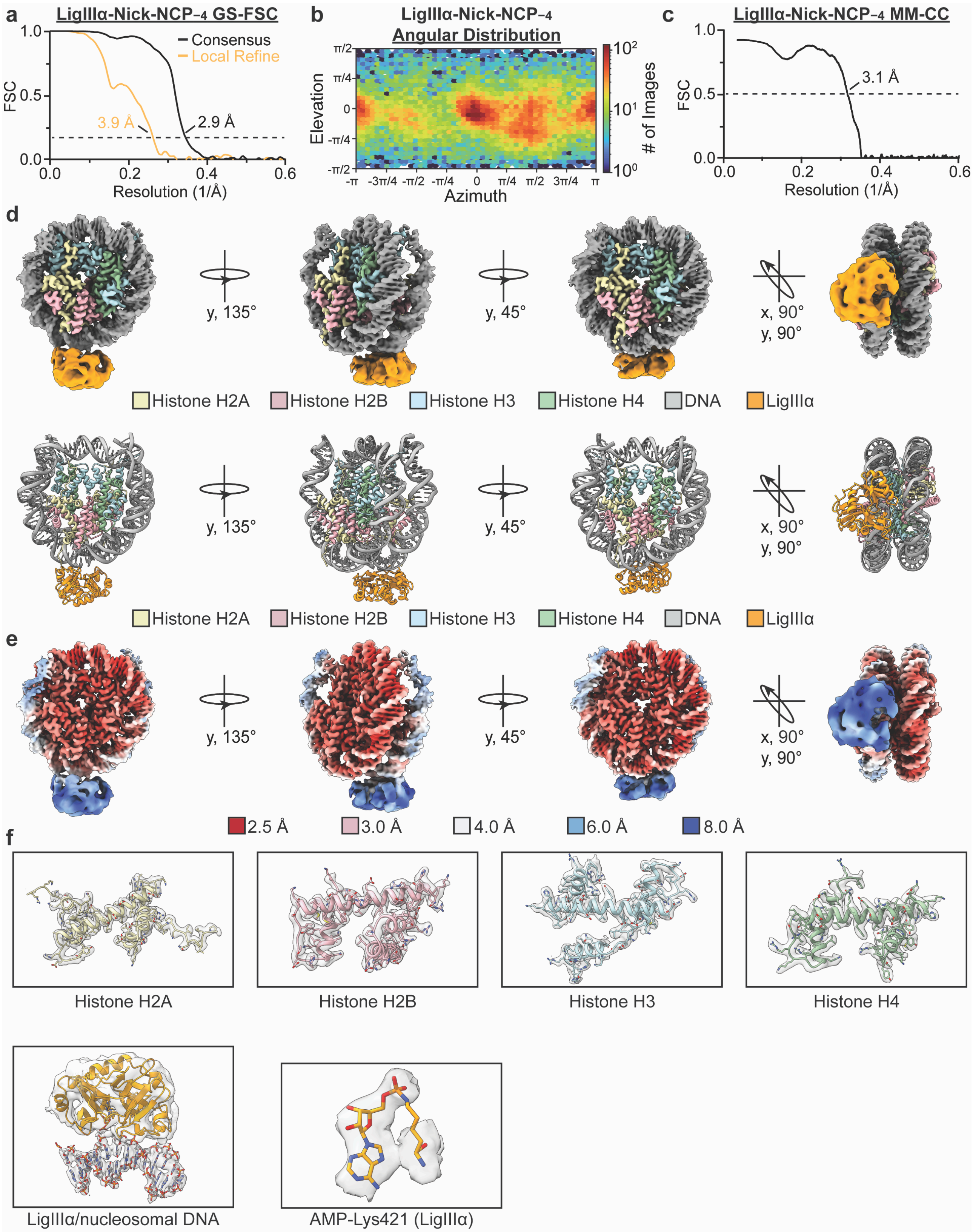

Supplementary Fig. 15: LigIIIα-Nick-NCP$_{-4}$ map and model quality assessment

a, Gold-standard Fourier shell correlation (GS-FSC) for the LigIIIα-Nick-NCP$_{-4}$ consensus cryo-EM map (solid black line) and the LigIIIα/nucleosomal DNA focus cryo-EM map (solid orange line). The dashed line corresponds to GS-FSC - 0.143. **b,** Angular distribution heatmap for the LigIIIα-Nick-NCP$_{-4}$ composite cryo-EM map. **c,** Map-to-model FSC for the LigIIIα-Nick-NCP$_{-4}$ model and composite cryo-EM map. The dashed line corresponds to FSC - 0.5. **d,** The final 2.9 Å LigIIIα-Nick-NCP$_{-4}$ composite cryo-EM map and model shown in four different orientations. **e**, Local resolution estimation for the LigIIIα-Nick-NCP$_{-4}$ composite cryo-EM map shown in four different orientations. **f,** Representative segmented densities for histones H2A, H2B, H3, H4, the LigIIIα/nucleosomal DNA binding interface, and the adenylylated-K421 from the LigIIIα-Nick-NCP$_{-4}$ composite cryo-EM map. The representative segmented densities from the cryo-EM map are shown as transparent gray surfaces. All source data in this figure are provided as a Source data file.

Supplementary Fig. 16: LigIIIα-Nick-NCP−2 map and model quality assessment

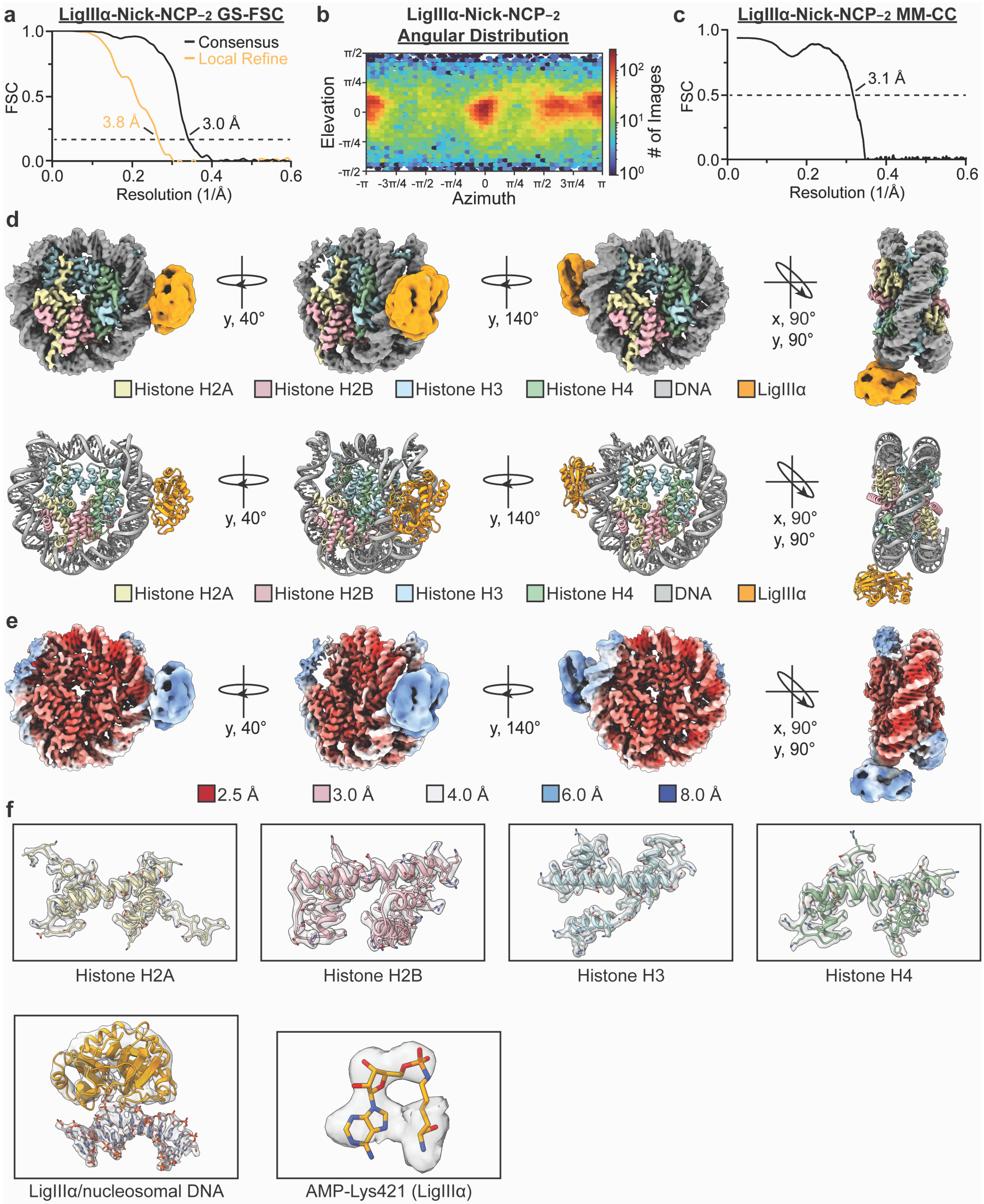

Supplementary Fig. 16: LigIIIα-Nick-NCP$_{-2}$ map and model quality assessment

a, Gold-standard Fourier shell correlation (GS-FSC) for the LigIIIα-Nick-NCP$_{-2}$ consensus cryo-EM map (solid black line) and the LigIIIα/nucleosomal DNA focus cryo-EM map (solid orange line). The dashed line corresponds to GS-FSC - 0.143. **b,** Angular distribution heatmap for the LigIIIα-Nick-NCP$_{-2}$ composite cryo-EM map. **c,** Map-to-model FSC for the LigIIIα-Nick-NCP$_{-2}$ model and composite cryo-EM map. The dashed line corresponds to FSC - 0.5. **d,** The final 3.0 Å LigIIIα-Nick-NCP$_{-2}$ composite cryo-EM map and model shown in four different orientations. **e**, Local resolution estimation for the LigIIIα-Nick-NCP$_{-2}$ composite cryo-EM map shown in four different orientations. **f,** Representative segmented densities for histones H2A, H2B, H3, H4, the LigIIIα/nucleosomal DNA binding interface, and the adenylylated-K421 from the LigIIIα-Nick-NCP$_{-2}$ composite cryo-EM map. The representative segmented densities from the cryo-EM map are shown as transparent gray surfaces. All source data in this figure are provided as a Source data file.

Supplementary Fig. 17: LigIIIα-Nick-NCP0 map and model quality assessment

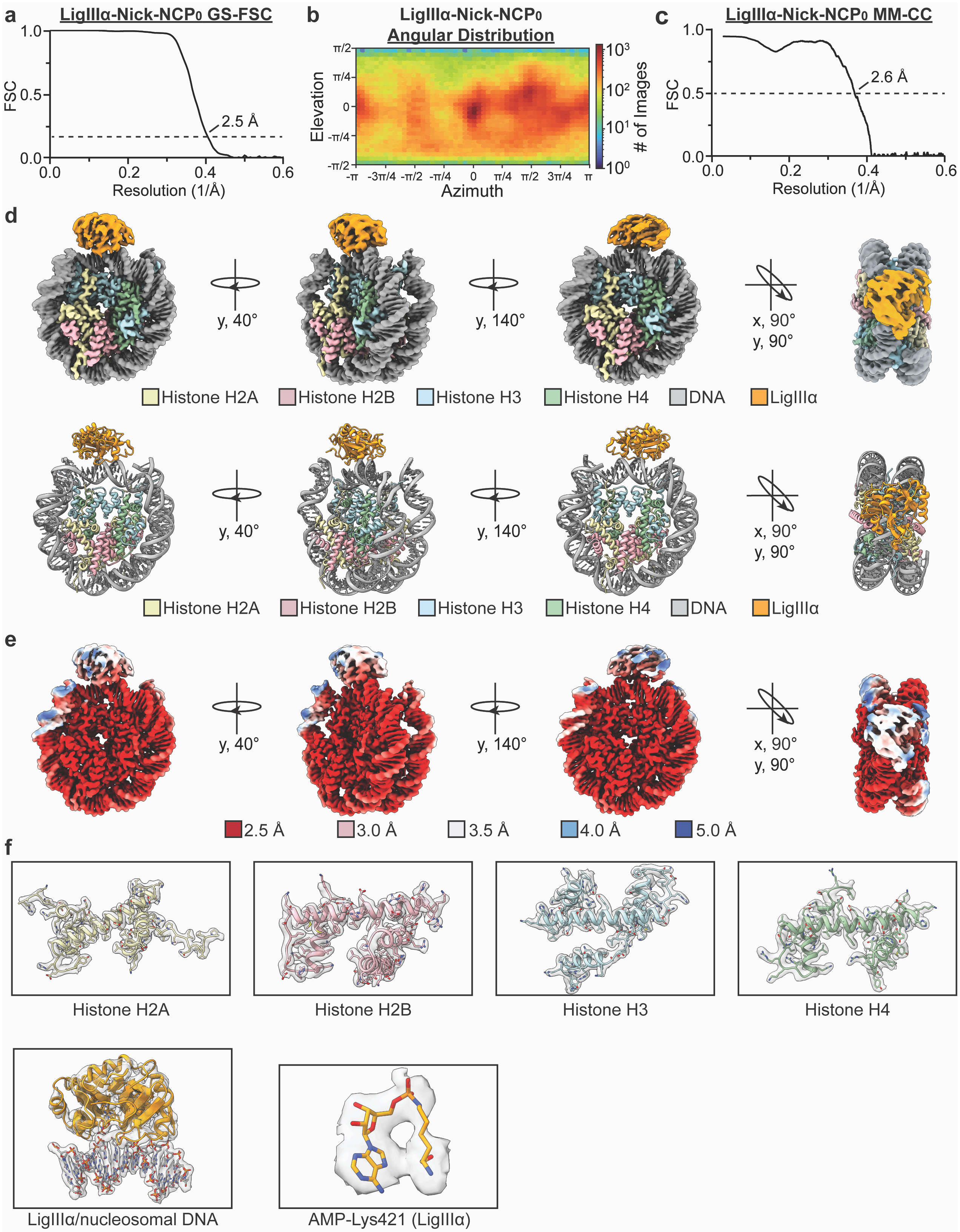

Supplementary Fig. 17: LigIIIα-Nick-NCP0 map and model quality assessment

a, Gold-standard Fourier shell correlation (GS-FSC) for the LigIIIα-Nick-NCP0 cryo-EM map (solid black line). The dashed line corresponds to GS-FSC - 0.143. **b,** Angular distribution heatmap for the LigIIIα-Nick-NCP0 cryo-EM map**. c,** Map-to-model FSC for the LigIIIα-Nick-NCP0 model and cryo-EM map. The dashed line corresponds to FSC - 0.5. **d,** The final 2.5 Å LigIIIα-Nick-NCP0 cryo-EM map and model shown in four different orientations. **e**, Local resolution estimation for the LigIIIα-Nick-NCP0 cryo-EM map shown in four different orientations. **f,** Representative segmented densities for histones H2A, H2B, H3, H4, the LigIIIα/nucleosomal DNA binding interface, and the adenylylated-K421 from the LigIIIα-Nick-NCP0 cryo-EM map. The representative segmented densities from the cryo-EM map are shown as transparent gray surfaces. All source data in this figure are provided as a Source data file.

Supplementary Fig. 18: Unassigned density in the LigIIIα-Nick-NCP cryo-EM reconstructions

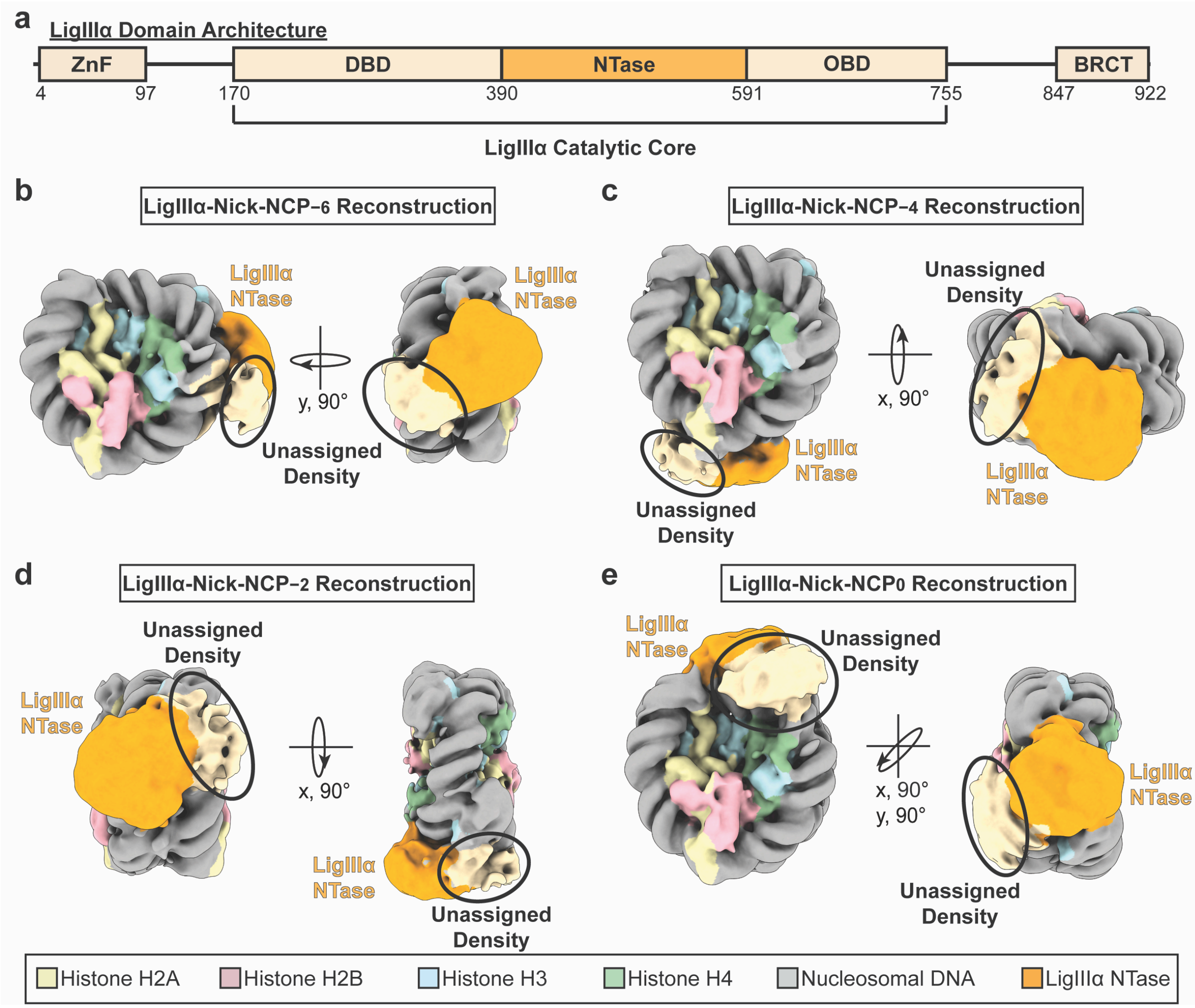

Supplementary Fig. 18: Unassigned density in the LigIIIα-Nick-NCP cryo-EM reconstructions

a, Diagram showing the domain architecture of LigIIIα. **b,** Low-pass filtered composite cryo-EM map of the LigIIIα-Nick-NCP$_{-6}$ complex. **c,** Low-pass filtered composite cryo-EM map of the LigIIIα-Nick-NCP$_{-4}$ complex. **d,** Low-pass filtered composite cryo-EM map of the LigIIIα-Nick-NCP$_{-2}$ complex. **e,** Low-pass filtered cryo-EM map of the LigIIIα-Nick-NCP$_{0}$ complex. For **b-e**, the density corresponding to the LigIIIα NTase domain is colored orange and the unassigned density is colored light orange.

Supplementary Fig. 19: Structural comparison of the LigIIIα/nucleosomal DNA binding interface at each nick position in the nucleosome

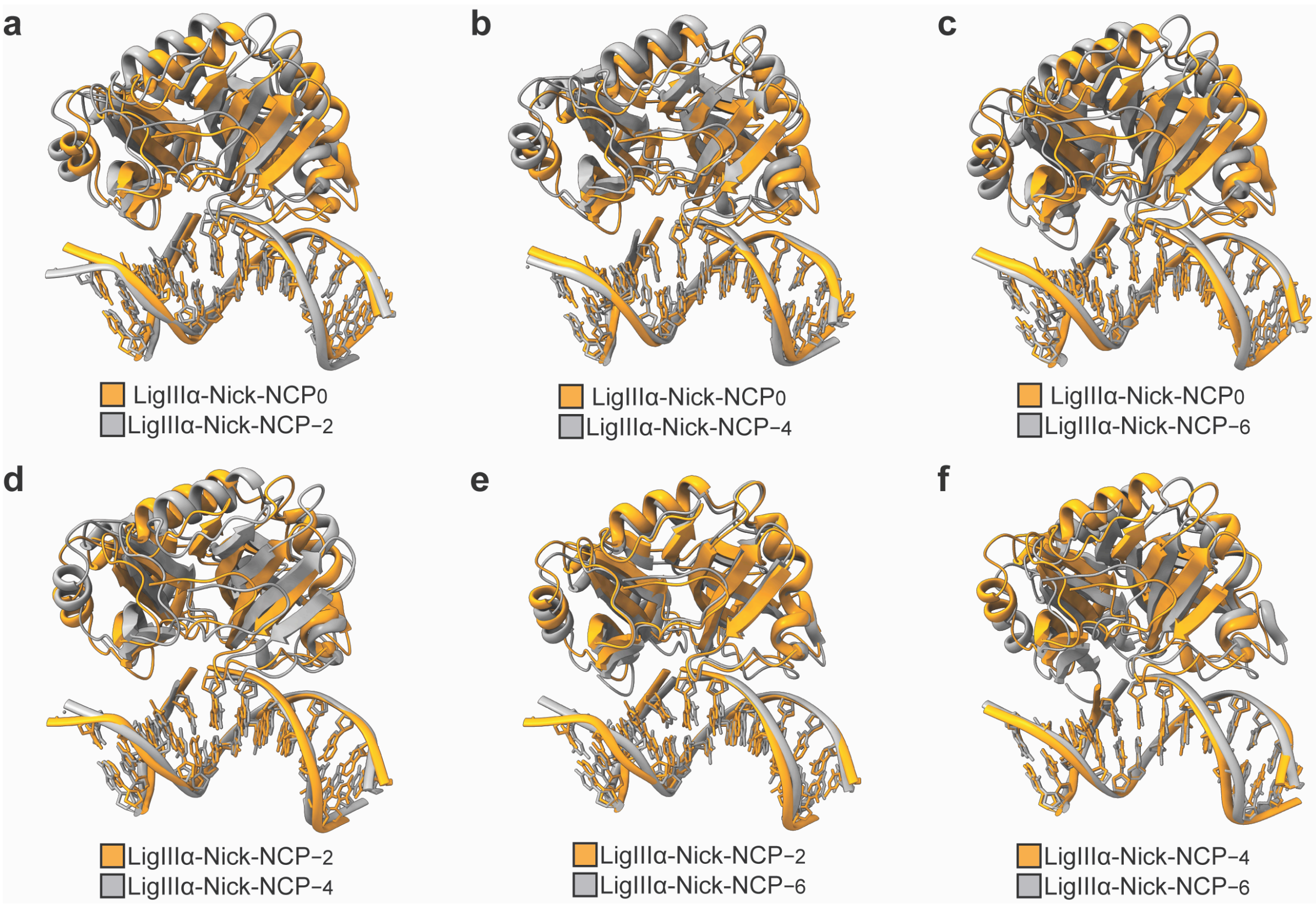

Supplementary Fig. 19: Structural comparison of the LigIIIα/nucleosomal DNA binding interface at each nick position in the nucleosome

a, Structural comparison of the LigIIIα/nucleosomal DNA binding interface in the LigIIIα-Nick-NCP$_{0}$ (orange) and LigIIIα-Nick-NCP$_{-2}$ (gray) complexes. **b,** Structural comparison of the LigIIIα/nucleosomal DNA binding interface in the LigIIIα-Nick-NCP$_{0}$ (orange) and LigIIIα-Nick-NCP$_{-4}$ (gray) complexes. **c,** Structural comparison of the LigIIIα/nucleosomal DNA binding interface in the LigIIIα-Nick-NCP$_{0}$ (orange) and LigIIIα-Nick-NCP$_{-6}$ (gray) complexes. **d,** Structural comparison of the LigIIIα/nucleosomal DNA binding interface in the LigIIIα-Nick-NCP$_{-2}$ (orange) and LigIIIα-Nick-NCP$_{-4}$ (gray) complexes. **e,** Structural comparison of the LigIIIα/nucleosomal DNA binding interface in the LigIIIα-Nick-NCP$_{-2}$ (orange) and LigIIIα-Nick-NCP$_{-6}$ (gray) complexes. **f,** Structural comparison of the LigIIIα/nucleosomal DNA binding interface in the LigIIIα-Nick-NCP$_{-4}$ (orange) and LigIIIα-Nick-NCP$_{-6}$ (gray) complexes.

Supplementary Fig. 20: LigIIIα adopts a catalytically inactive conformation during nick recognition in the nucleosome.

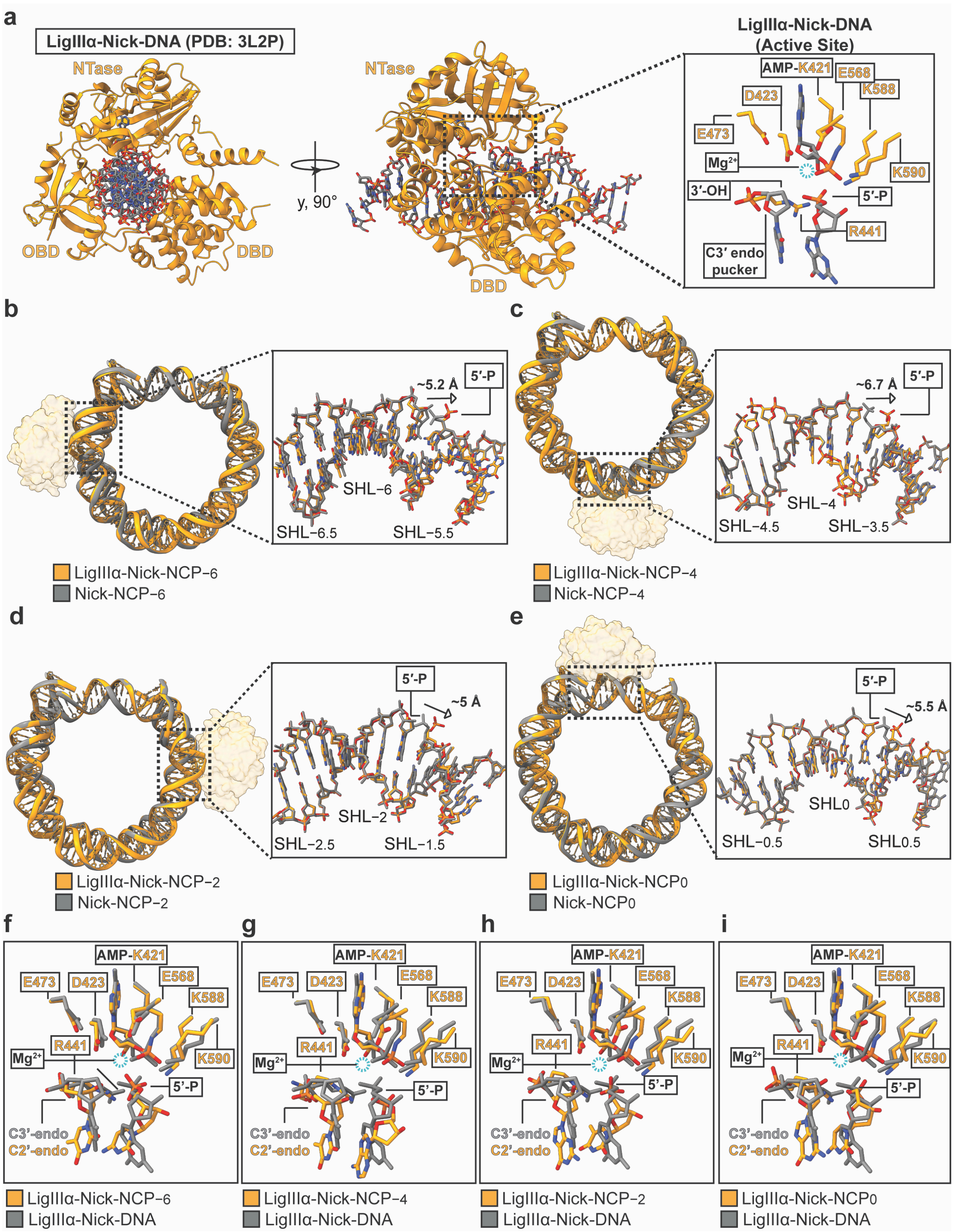

Supplementary Fig. 20: LigIIIα adopts a catalytically inactive conformation during nick recognition in the nucleosome.

a, A model of the LigIIIα-Nick-DNA complex (PDB: 3L2P) shown in two orientations, with a focused view of the LigIIIα-active site shown as an inset. The unoccupied Mg^{2+} binding site is denoted with a dashed blue circle. **b,** Structural comparison of the nucleosomal DNA in the Nick-NCP$_{-6}$ (gray) and LigIIIα-Nick-NCP$_{-6}$ (orange) structures. The inset is rotated 180°. **c,** Structural comparison of the nucleosomal DNA in the Nick-NCP$_{-4}$ (gray) and LigIIIα-Nick-NCP$_{-4}$ (orange) structures. The inset is rotated 180°. **d,** Structural comparison of the nucleosomal DNA in the Nick-NCP$_{-2}$ (gray) and LigIIIα-Nick-NCP$_{-2}$ (orange) structures. The inset is rotated 180°. **e,** Structural comparison of the nucleosomal DNA in the Nick-NCP$_{0}$ (gray) and LigIIIα-Nick-NCP$_{0}$ (orange) structures. The inset is rotated 180°. In **b-e**, LigIIIα is shown as a transparent orange surface. **f,** Focused view of the LigIIIα active site in the LigIIIα-Nick-DNA (gray) and LigIIIα-Nick-NCP$_{-6}$ complex (orange). **g,** Focused view of the LigIIIα active site in the LigIIIα-Nick-DNA (gray) and LigIIIα-Nick-NCP$_{-4}$ complex (orange). **h,** Focused view of the LigIIIα active site in the LigIIIα-Nick-DNA (gray) and LigIIIα-Nick-NCP$_{-2}$ complex (orange). **i,** Focused view of the LigIIIα active site in the LigIIIα-Nick-DNA (gray) and LigIIIα-Nick-NCP$_{0}$ complex (orange). In **f-i**, the unoccupied Mg^{2+} binding site is denoted with a dashed blue circle.

Supplementary Fig. 21: Single-turnover kinetic analysis of XRCC1-LigIIIα nick ligation in the nucleosome

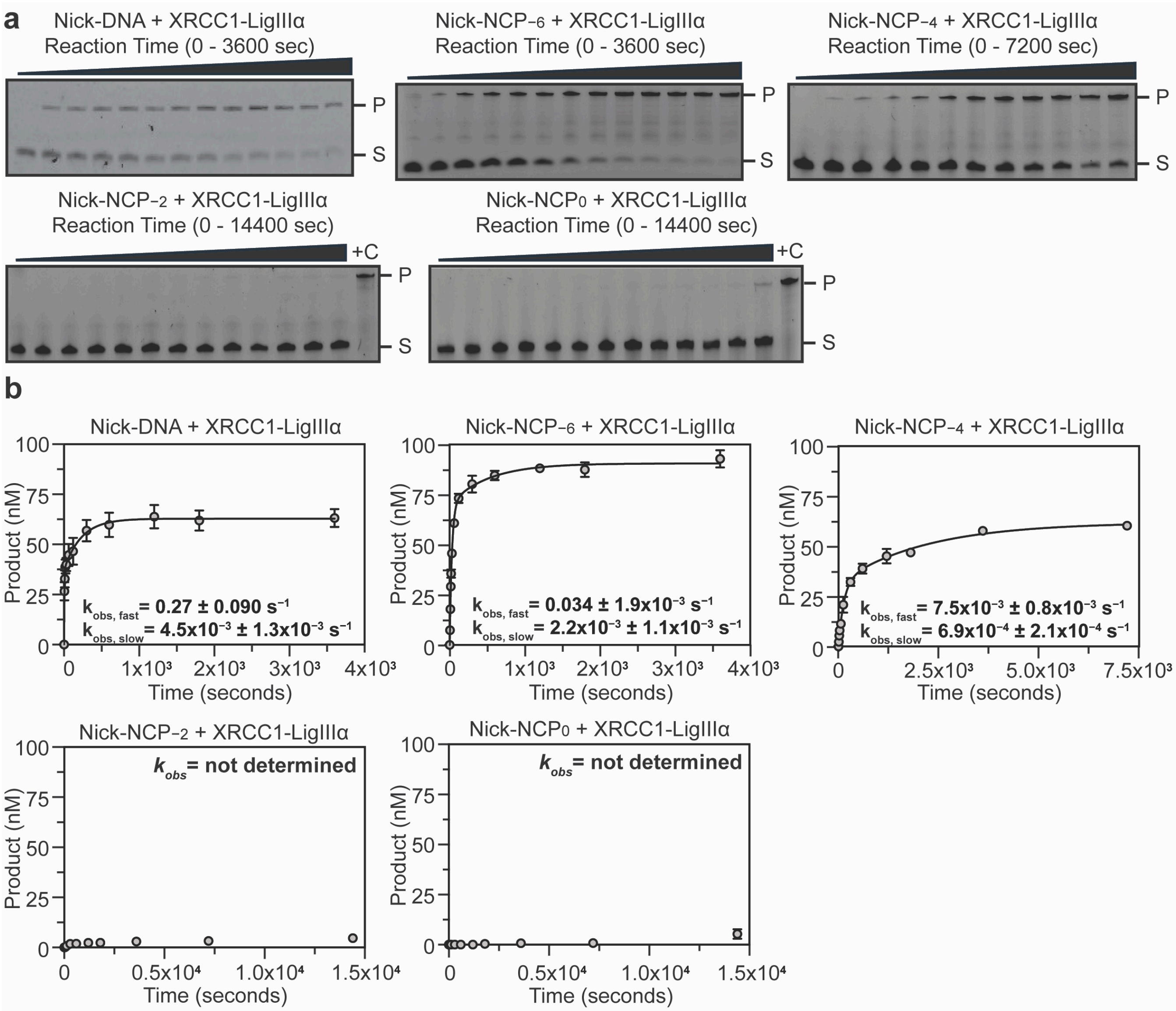

Supplementary Fig. 21: Single-turnover kinetic analysis of XRCC1-LigIIIα nick ligation in the nucleosome

a, Representative denaturing Urea-PAGE gels from the single-turnover kinetic experiments for XRCC1-LigIIIα and Nick-DNA, Nick-NCP0, Nick-NCP−2, Nick-NCP−4, and Nick-NCP−6. The substrate (S, Nick-DNA or Nick-NCP) and product (P, ligated DNA or NCP) were detected using the 6-FAM label on the nucleosomal DNA of each NCP. A 147 nt single-stranded DNA was loaded as a positive control (+C) for experiments with less than 10% product formed over the kinetic time course. The gels are representative of three independent single-turnover kinetic experiments performed for XRCC1-LigIIIα and the Nick-DNA and each Nick-NCP. **b,** Quantification of the single-turnover kinetic experiments for XRCC1-LigIIIα and Nick-DNA, Nick-NCP−6, Nick-NCP−4, Nick-NCP−2, and Nick-NCP0. The data points represent the mean ± standard deviation from three independent replicate experiments. The error bars are included for all experimental data points, but some error bars are smaller than the circles used to represent data points. The ligation rate (k_{obs}) is shown as an inset for each experiment and represents the mean ± standard error of the mean from the three independent replicate experiments. The ligation rate (k_{obs}) was not determined for experiments with less than 10% product formed over the kinetic time course. All source data in this figure are provided as a Source Data file.

Supplementary Fig. 22: EMSA Analysis of the XRCC1-LigIIIα-Nick-NCP interaction

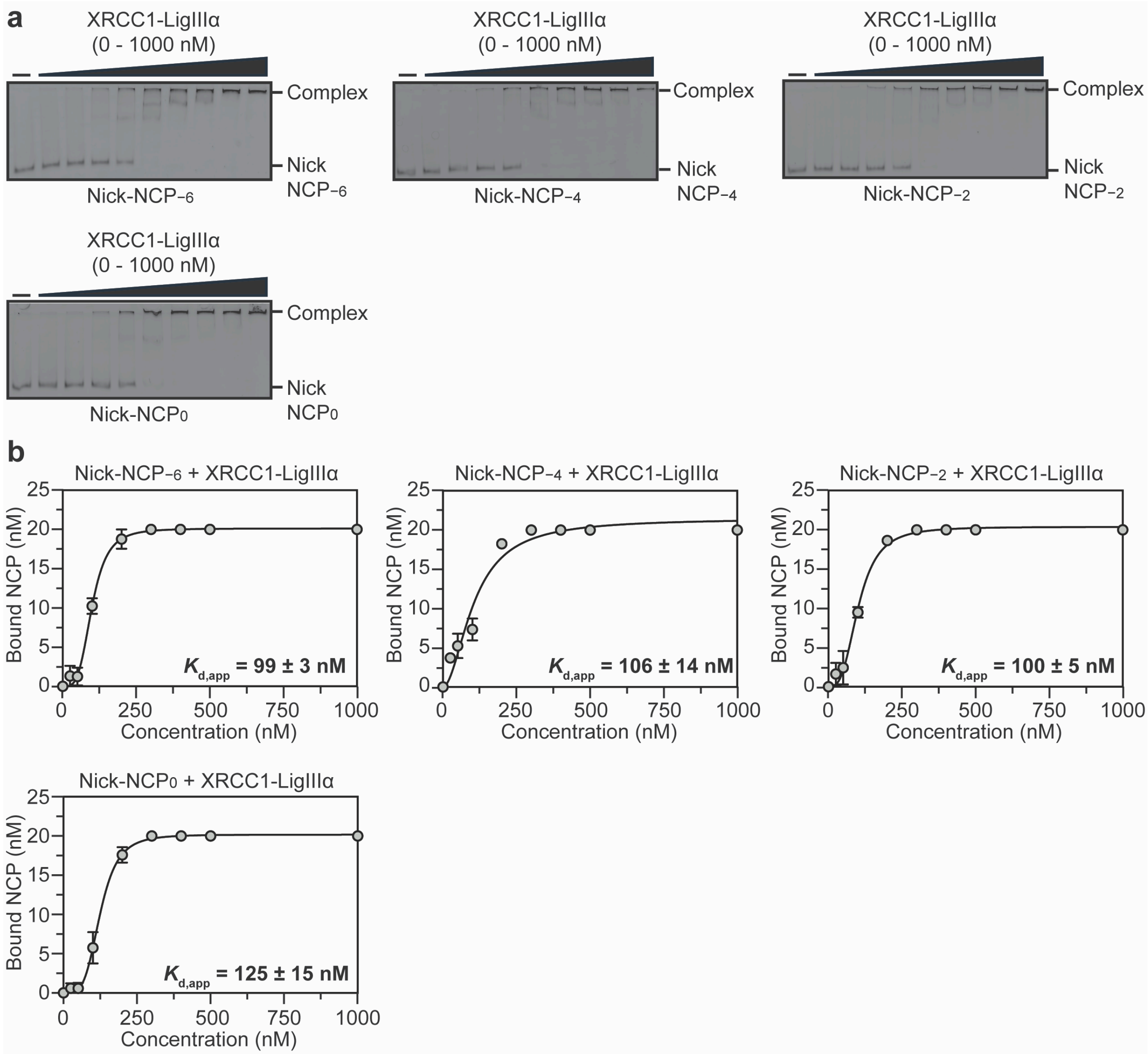

Supplementary Fig. 22: EMSA Analysis of the XRCC1-LigIIIα-Nick-NCP interaction

a, Representative native PAGE gels from the electrophoretic mobility shift assays (EMSAs) of XRCC1-LigIIIα and Nick-NCP$_{-6}$, Nick-NCP$_{-4}$, Nick-NCP$_{-2}$, and Nick-NCP$_{0}$. The free Nick-NCP and XRCC1-LigIIIα-Nick-NCP complex were detected using the 6-FAM label on the nucleosomal DNA of each NCP. The gels are representative of three independent EMSA experiments performed for XRCC1-LigIIIα and each Nick-NCP. **b,** Quantification of the EMSA experiments for XRCC1-LigIIIα and Nick-NCP$_{-6}$, Nick-NCP$_{-4}$, Nick-NCP$_{-2}$, and Nick-NCP$_{0}$. The data points represent the mean ± standard deviation from the three independent replicate experiments. The error bars are included for all experimental data points, but some error bars are smaller than the circles used to represent data points. The apparent binding affinity ($K_{d,app}$) is shown as an inset for each experiment and represents the mean ± standard deviation from the three independent replicate experiments. All source data in this figure are provided as a Source Data file.

Supplementary Fig. 23: XRCC1-LigIIIα-Nick-NCP−6 single particle analysis processing workflow

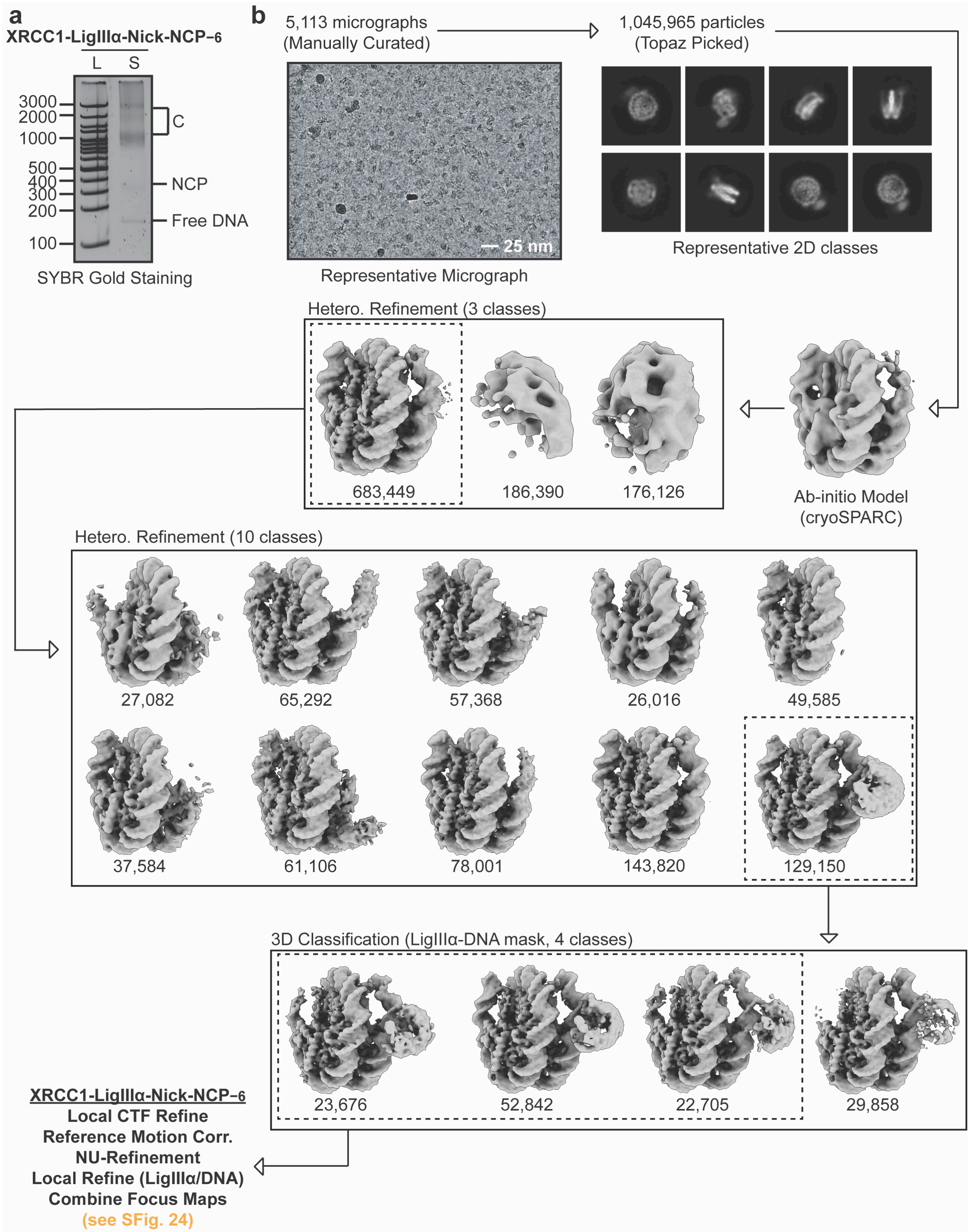

Supplementary Fig. 23: XRCC1-LigIIIα-Nick-NCP−6 single particle analysis processing workflow

a, Native PAGE gel of the XRCC1-LigIIIα-Nick-NCP−6 cryo-EM sample (S) and a 100 bp DNA ladder (L). The NCP−6 and XRCC1-LigIIIα-Nick-NCP−6 complex were visualized with SYBR gold staining. The bands corresponding to free DNA, the NCP, and XRCC1-LigIIIα-Nick-NCP−6 (C) are labeled. **b,** Cryo-EM data processing workflow for the XRCC1-LigIIIα-Nick-NCP−6 dataset. A representative micrograph (n=5,113) and representative 2D classes from the XRCC1-LigIIIα-Nick-NCP−6 dataset are shown. The maps chosen for further classification and/or refinement throughout the data processing pipeline are boxed. The final map, final model, and quality assessment metrics for XRCC1-LigIIIα-Nick-NCP−6 can be found in Supplementary Fig. 24. All source data in this figure are provided as a Source data file.

Supplementary Fig. 24: XRCC1-LigIIIα-Nick-NCP−6 map and model quality assessment

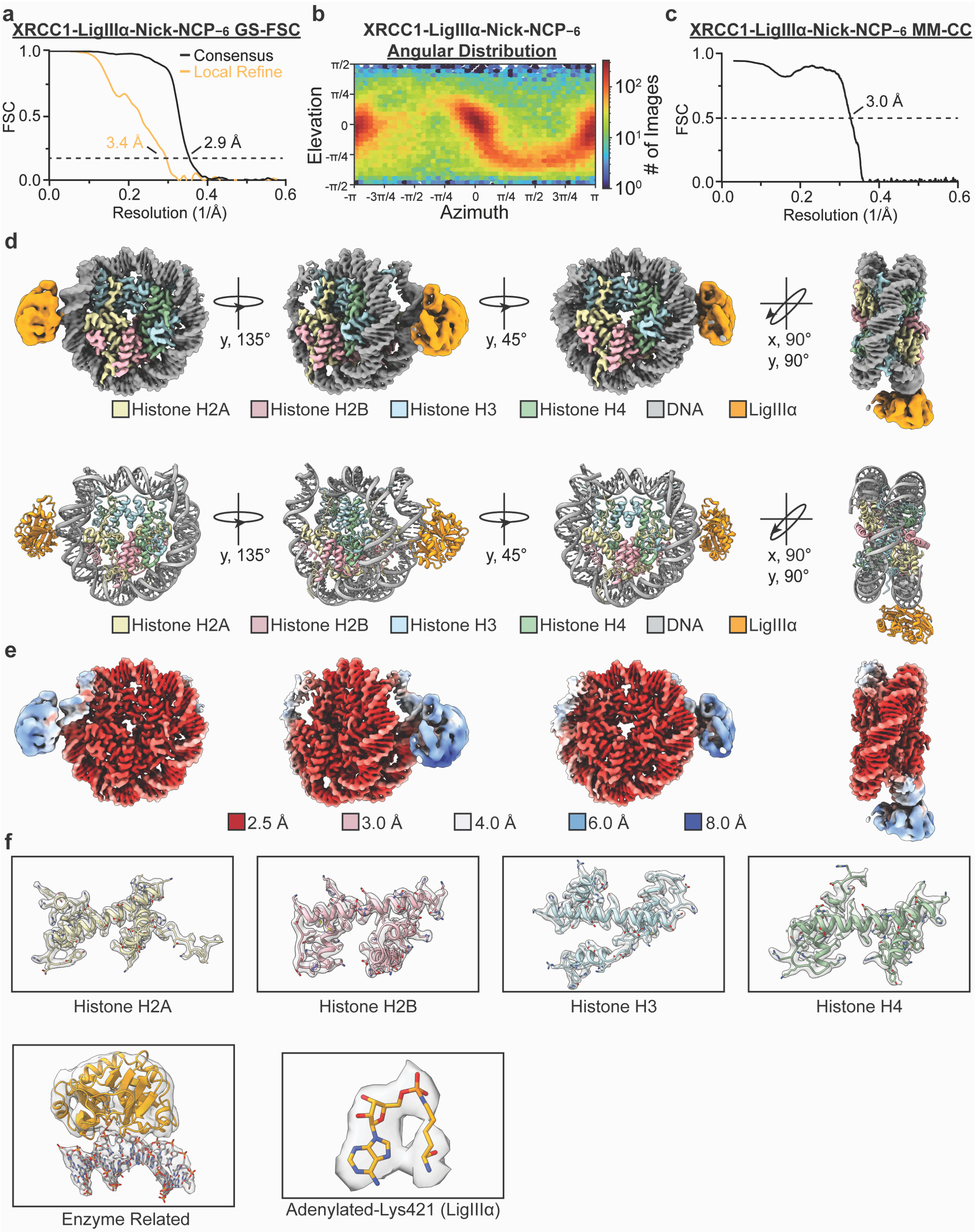

Supplementary Fig. 24: XRCC1-LigIIIα-Nick-NCP$_{-6}$ map and model quality assessment

a, Gold-standard Fourier shell correlation (GS-FSC) for the XRCC1-LigIIIα-Nick-NCP$_{-6}$ consensus cryo-EM map (solid black line) and the LigIIIα/nucleosomal DNA focus cryo-EM map (solid orange line). The dashed line corresponds to GS-FSC - 0.143. **b,** Angular distribution heatmap for the XRCC1-LigIIIα-Nick-NCP$_{-6}$ composite cryo-EM map. **c,** Map-to-model FSC for the XRCC1-LigIIIα-Nick-NCP$_{-6}$ model and composite cryo-EM map. The dashed line corresponds to FSC - 0.5. **d,** The final 2.9 Å XRCC1-LigIIIα-Nick-NCP$_{-6}$ composite cryo-EM map and model shown in four different orientations. **e**, Local resolution estimation for the XRCC1-LigIIIα-Nick-NCP$_{-6}$ composite cryo-EM map shown in four different orientations. **f,** Representative segmented densities for histones H2A, H2B, H3, H4, the LigIIIα/nucleosomal DNA binding interface, and the adenylylated-K421 from the XRCC1-LigIIIα-Nick-NCP$_{-6}$ composite cryo-EM map. The representative segmented densities from the cryo-EM map are shown as transparent gray surfaces. All source data in this figure are provided as a Source data file.

Supplementary Table 1: LigIIIα and XRCC1-LigIIIα apparent nucleosome binding affinities and ligation rates

Protein	Substrate	k_{obs} (fast, s^{-1})	Fold Change (Nick-DNA)	k_{obs} (slow, s^{-1})	Fold Change (Nick-DNA)	$K_{d,app}$ (nM)	Hill Coeff.
LigIIIα	Nick-DNA	0.21 ± 0.027	-	$6.1\times10^{-3} \pm 2.0\times10^{-3}$	-	NT	-
	Nick-NCP−6	$0.061 \pm 5.6\times10^{-3}$	3.4	$2.4\times10^{-3} \pm 1.3\times10^{-3}$	2.5	120 ± 5	2.7
	Nick-NCP−4	$6.8\times10^{-3} \pm 1.0\times10^{-3}$	31	$8.1\times10^{-4} \pm 2.2\times10^{-4}$	7.5	135 ± 8	2.6
	Nick-NCP−2	ND	-	ND	-	106 ± 11	1.5
	Nick-NCP0	ND	-	ND	-	134 ± 2	2.1
	5S-Nick-NCP−2	ND	-	ND	-	NT	-
	5S-Nick-NCP0	ND	-	ND	-	NT	-
XRCC1-LigIIIα	Nick-DNA	0.27 ± 0.090	-	$4.5\times10^{-3} \pm 1.3\times10^{-3}$	-	-	-
	Nick-NCP−6	$0.034 \pm 1.9\times10^{-3}$	7.9	$2.2\times10^{-3} \pm 1.1\times10^{-3}$	2	99 ± 3	3.8
	Nick-NCP−4	$7.5\times10^{-3} \pm 0.8\times10^{-3}$	36	$6.9\times10^{-4} \pm 2.1\times10^{-4}$	6.5	106 ± 14	1.8
	Nick-NCP−2	ND	-	ND	-	100 ± 5	3.0
	Nick-NCP0	ND	-	ND	-	125 ± 15	4.0

ND – Not determined due to minimal product formation during the single-turnover kinetic time course

NT – Experiments not performed

Supplementary Table 2: Cryo-EM data collection, refinement, and validation

**Data collection and processing**	
**Dataset**	**ND-NCP**
Magnification	130,000x
Voltage (kV)	300
Electron exposure ($e^-/Å^2$)	50
Defocus range (μm)	−1.2 to −1.8
Pixel size (Å)	0.652
Symmetry imposed	C1
Initial particle number	1,475,651
**Structure**	**ND-NCP**
Final particle number	439,555
Map resolution (Å)	2.6
FSC threshold	0.143
PDB accession	**10XZ**
EMDB accession	**EMD-75521**
**Refinement**	
Initial model used (PDB ID)	9DWF
Model resolution (Å)	2.6
FSC threshold	0.5
**Model composition**	
Nonhydrogen atoms	12,043
Protein residues	756
Nucleotide	294
**B factors ($Å^2$)**	
Protein	87.05
Nucleotide	135.10
**r.m.s. deviations**	
Bond Length (Å) (# > 4σ)	0.005 (0)
Bond Angles (°) (# > 4σ)	0.803 (0)
**Validation**	
MolProbity score	1.17
Clashscore	3.78
Rotamer Outliers (%)	0.32
**Ramachandran plot**	
Favored (%)	98.51
Allowed (%)	1.49
Disallowed (%)	0.00

Supplementary Table 3: Cryo-EM data collection, refinement, and validation

**Data collection and processing**				
**Dataset**	**Nick-NCP$_{-4}$**		**Nick-NCP$_{-6}$**	
Magnification	130,000x		130,000x	
Voltage (kV)	300		300	
Electron exposure ($e^{-}/Å^{2}$)	50		50	
Defocus range (μm)	−1.2 to −1.8		−1.2 to −1.8	
Pixel size (Å)	0.652		0.652	
Symmetry imposed	C1		C1	
Initial particle number	573,461		629,703	
**Structure**	**Nick-NCP$_{-4}$**	**LigIIIα-Nick-NCP$_{-4}$**	**Nick-NCP$_{-6}$**	**LigIIIα-Nick-NCP$_{-6}$**
Final particle number	97,160	39,115	72,441	74,600
Map resolution (Å)	2.7	2.9	2.8	2.8
FSC threshold	0.143	0.143	0.143	0.143
PDB accession	**10YC**	**10YG**	**10YD**	**10YH**
EMDB accession	**EMD-75524**	**EMD-75528**<br>**EMD-75533**<br>**EMD-75534**	**EMD-75525**	**EMD-75529**<br>**EMD-75535**<br>**EMD-75536**
**Refinement**				
Initial model used (PDB ID)	9DWF	9DWF, 3L2P	9DWF	9DWF, 3L2P
Model resolution (Å)	2.8	3.1	2.9	3.0
FSC threshold	0.5	0.5	0.5	0.5
**Model composition**				
Nonhydrogen atoms	11,998	13,681	11,959	13,642
Protein residues	753	961	748	956
Nucleotide	294	294	294	294
**B factors ($Å^2$)**				
Protein	89.08	109.39	83.94	112.74
Nucleotide	151.07	159.02	138.59	142.41
**r.m.s. deviations**				
Bond Length (Å) (# > 4σ)	0.004 (0)	0.005 (0)	0.005 (0)	0.008 (0)
Bond Angles (°) (# > 4σ)	0.778 (0)	0.812 (0)	0.785 (1)	0.828 (0)
**Validation**				
MolProbity score	1.12	1.17	1.19	1.20
Clashscore	3.24	3.77	4.00	3.38
Rotamer Outliers (%)	0.16	0.37	0.32	0.25
**Ramachandran plot**				
Favored (%)	98.24	97.99	98.22	97.65
Allowed (%)	1.76	2.01	1.78	2.35
Disallowed (%)	0.00	0.00	0.00	0.00

Supplementary Table 4: Cryo-EM data collection, refinement, and validation

**Data collection and processing**				
**Dataset**	**Nick-NCP$_0$**		**Nick-NCP$_{-2}$**	
Magnification	130,000x		130,000x	
Voltage (kV)	300		300	
Electron exposure (e^{-}/Å^2)	50		50	
Defocus range (μm)	−1.2 to −1.8		−1.2 to −1.8	
Pixel size (Å)	0.652		0.652	
Symmetry imposed	C1		C1	
Initial particle number	1,476,778		510,094	
**Structure**	**Nick-NCP$_0$**	**LigIIIα-Nick-NCP$_0$**	**Nick-NCP$_{-2}$**	**LigIIIα-Nick-NCP$_{-2}$**
Final particle number	141,040	463,243	50,482	50,548
Map resolution (Å)	2.8	2.5	2.9	3.0
FSC threshold	0.143	0.143	0.143	0.143
PDB accession	**10YA**	**10YE**	**10YB**	**10YF**
EMDB accession	**EMD-75522**	**EMD-75526**	**EMD-75523**	**EMD-75527**<br>**EMD-75531**<br>**EMD-75532**
**Refinement**				
Initial model used (PDB ID)	9DWF	9DWF, 3L2P	9DWF	9DWF, 3L2P
Model resolution (Å)	2.8	2.6	3.0	3.1
FSC threshold	0.5	0.5	0.5	0.5
**Model composition**				
Nonhydrogen atoms	11,998	13,681	11,904	13,644
Protein residues	753	961	742	957
Nucleotide	294	294	294	294
**B factors (Å^2)**				
Protein	59.75	110.23	87.64	99.00
Nucleotide	105.09	129.17	154.24	151.30
**r.m.s. deviations**				
Bond Length (Å) (# > 4σ)	0.004 (0)	0.005 (0)	0.005 (0)	0.007 (0)
Bond Angles (°) (# > 4σ)	0.772 (0)	0.802 (0)	0.810 (1)	0.813 (0)
**Validation**				
MolProbity score	1.19	1.17	1.16	1.17
Clashscore	4.08	3.81	3.69	3.82
Rotamer Outliers (%)	0.16	0.25	0.49	0.37
**Ramachandran plot**				
Favored (%)	98.91	98.41	98.62	98.08
Allowed (%)	1.09	1.59	1.38	1.92
Disallowed (%)	0.00	0.00	0.00	0.00

Supplementary Table 5: Cryo-EM data collection, refinement, and validation

**Data collection and processing**	
**Dataset**	**XRCC1-LigIIIα-Nick-NCP$_{-6}$**
Magnification	130,000x
Voltage (kV)	300
Electron exposure (e$^-$/Å^2)	50
Defocus range (μm)	−1.2 to −1.8
Pixel size (Å)	0.652
Symmetry imposed	C1
Initial particle number	599,935
**Structure**	**XRCC1-LigIIIα-Nick-NCP$_{-6}$**
Final particle number	99,223
Map resolution (Å)	2.9
FSC threshold	0.143
PDB accession	**10YI**
EMDB accession	**EMD-75530**<br>**EMD-75537**<br>**EMD-75538**
**Refinement**	
Initial model used (PDB ID)	9DWF, 3L2P
Model resolution (Å)	3.0
FSC threshold	0.5
**Model composition**	
Nonhydrogen atoms	13642
Protein residues	956
Nucleotide	294
**B factors (Å^2)**	
Protein	95.17
Nucleotide	137.49
**r.m.s. deviations**	
Bond Length (Å) (# > 4σ)	0.005 (0)
Bond Angles (°) (# > 4σ)	0.791 (0)
**Validation**	
MolProbity score	1.25
Clashscore	3.34
Rotamer Outliers (%)	0.37
**Ramachandran plot**	
Favored (%)	97.33
Allowed (%)	2.67
Disallowed (%)	0.00

Supplementary Table 6: Oligonucleotides for generating recombinant 601 nucleosomes

Oligo	Sequence (5′ – 3′)
**ND-NCP**	
Oligo 1	ATCGAGAATCCCGGTGCCGAGGCCGCTCAATTGGTCGTAGACAGCTCTAGCACCG<br>CTTAAACGCACGTACGCGCTGTCCCCCGCGTTTTAACCGCCAAGGGGATTACTCCC<br>TAGTCTCCAGGCACGTGTCAGATATATACATCCGAT
Oligo 2	/6FAM/ATCGGATGTATATATCTGACACGTGCCTGGAGACTAGGGAGTAATCCCCTT<br>GGCGGTTAAAACGCGGGGGACAGCGCGTACGTGCGTTTAAGCGGTGCTAGAGCTG<br>TCTACGACCAATTGAGCGGCCTCGGCACCGGGATTCTCGAT
**Nick-NCP$_{-6}$**	
Oligo 1	ATCGAGAATCCCGGTGCCGAGGCCGCTCAATTGGTCGTAGACAGCTCTAGCACCG<br>CTTAAACGCACGTACGCGCTGTCCCCCGCGTTTTAACCGCCAAGGGGATTACTCCC<br>TAGTCTCCAGGCACGTGTCAGATATATACATCCGAT
Oligo 2	/6FAM/ATCGGATGTATATAT
Oligo 3	/5Phos/CTGACACGTGCCTGGAGACTAGGGAGTAATCCCCTTGGCGGTTAAAACGC<br>GGGGGACAGCGCGTACGTGCGTTTAAGCGGTGCTAGAGCTGTCTACGACCAATTG<br>AGCGGCCTCGGCACCGGGATTCTCGAT
**Nick-NCP$_{-4}$**	
Oligo 1	ATCGAGAATCCCGGTGCCGAGGCCGCTCAATTGGTCGTAGACAGCTCTAGCACCG<br>CTTAAACGCACGTACGCGCTGTCCCCCGCGTTTTAACCGCCAAGGGGATTACTCCC<br>TAGTCTCCAGGCACGTGTCAGATATATACATCCGAT
Oligo 2	/6FAM/ATCGGATGTATATATCTGACACGTGCCTGGAGACT
Oligo 3	/5Phos/AGGGAGTAATCCCCTTGGCGGTTAAAACGCGGGGGACAGCGCGTACGTGC<br>GTTTAAGCGGTGCTAGAGCTGTCTACGACCAATTGAGCGGCCTCGGCACCGGGAT<br>TCTCGAT
**Nick-NCP$_{-2}$**	
Oligo 1	ATCGAGAATCCCGGTGCCGAGGCCGCTCAATTGGTCGTAGACAGCTCTAGCACCG<br>CTTAAACGCACGTACGCGCTGTCCCCCGCGTTTTAACCGCCAAGGGGATTACTCCC<br>TAGTCTCCAGGCACGTGTCAGATATATACATCCGAT
Oligo 2	/6FAM/ATCGGATGTATATATCTGACACGTGCCTGGAGACTAGGGAGTAATCCCCTT<br>GGCGG
Oligo 3	/5Phos/TTAAAACGCGGGGGACAGCGCGTACGTGCGTTTAAGCGGTGCTAGAGCTG<br>TCTACGACCAATTGAGCGGCCTCGGCACCGGGATTCTCGAT
**Nick-NCP$_{0}$**	
Oligo 1	ATCGAGAATCCCGGTGCCGAGGCCGCTCAATTGGTCGTAGACAGCTCTAGCACCG<br>CTTAAACGCACGTACGCGCTGTCCCCCGCGTTTTAACCGCCAAGGGGATTACTCCC<br>TAGTCTCCAGGCACGTGTCAGATATATACATCCGAT
Oligo 2	/6FAM/ATCGGATGTATATATCTGACACGTGCCTGGAGACTAGGGAGTAATCCCCTT<br>GGCGGTTAAAACGCGGGGGACAGCG
Oligo 3	/5Phos/CGTACGTGCGTTTAAGCGGTGCTAGAGCTGTCTACGACCAATTGAGCGGCC<br>TCGGCACCGGGATTCTCGAT

*Nucleosomes used for cryo-EM were generated with oligonucleotides lacking the 6FAM label.

Supplementary Table 7: Oligonucleotides for generating recombinant *5S* nucleosomes

Oligo	Sequence (5′ – 3′)
***5S*-Nick-NCP$_{-2}$**	
Oligo 1	GCTTGTTTTCCTGCCTGGGGGAAAAGACCCTGGCATGGGGAGGAGCTGGGCCCCC<br>CCCAGAAGGCAGCACAAGGGGAGGAAAAGTCAGCCTTGTGCTCGCCTACGGCCAT<br>ACCACCCTGAAAGTGCCCGATATCGTCTGATCTCGGA
Oligo 2	**/6FAM/**TCCGAGATCAGACGATATCGGGCACTTTCAGGGTGGTATGGCCGTAGGCG<br>AGCACA
Oligo 3	**/5Phos/**AGGCTGACTTTTCCTCCCCTTGTGCTGCCTTCTGGGGGGGCCCAGCTCCTC<br>CCCATGCCAGGGTCTTTTCCCCCAGGCAGGAAAACAAGC
***5S*-Nick-NCP$_{0}$**	
Oligo 1	GCTTGTTTTCCTGCCTGGGGGAAAAGACCCTGGCATGGGGAGGAGCTGGGCCCCC<br>CCCAGAAGGCAGCACAAGGGGAGGAAAAGTCAGCCTTGTGCTCGCCTACGGCCAT<br>ACCACCCTGAAAGTGCCCGATATCGTCTGATCTCGGA
Oligo 2	**/6FAM/**TCCGAGATCAGACGATATCGGGCACTTTCAGGGTGGTATGGCCGTAGGCG<br>AGCACAAGGCTGACTTTTCCTCCCCT
Oligo 3	**/5Phos/**TGTGCTGCCTTCTGGGGGGGCCCAGCTCCTCCCCATGCCAGGGTCTTTTCC<br>CCCAGGCAGGAAAACAAGC
