# Supplementary material for: Molecular basis of nick ligation in the nucleosome by DNA Ligase IIIα
Source: Nat Commun. 2026 Sep 2;17:10460. doi: 10.1038/s41467-026-77207-7 (PMC13635136; doi:10.1038/s41467-026-77207-7)
Supplement: Supplementary file 2 [file 41467_2026_77207_MOESM2_ESM.pdf]

nature portfolio

Corresponding author(s): Tyler M. Weaver, Ph.D.

Last updated by author(s): 7/17/2026

Reporting Summary

Nature Portfolio wishes to improve the reproducibility of the work that we publish. This form provides structure for consistency and transparency in reporting. For further information on Nature Portfolio policies, see our Editorial Policies and the Editorial Policy Checklist.

Statistics

For all statistical analyses, confirm that the following items are present in the figure legend, table legend, main text, or Methods section.

n/a	Confirmed	
☐	☒	The exact sample size (*n*) for each experimental group/condition, given as a discrete number and unit of measurement
☒	☐	A statement on whether measurements were taken from distinct samples or whether the same sample was measured repeatedly
☐	☒	The statistical test(s) used AND whether they are one- or two-sided<br>*Only common tests should be described solely by name; describe more complex techniques in the Methods section.*
☒	☐	A description of all covariates tested
☒	☐	A description of any assumptions or corrections, such as tests of normality and adjustment for multiple comparisons
☐	☒	A full description of the statistical parameters including central tendency (e.g. means) or other basic estimates (e.g. regression coefficient) AND variation (e.g. standard deviation) or associated estimates of uncertainty (e.g. confidence intervals)
☐	☒	For null hypothesis testing, the test statistic (e.g. *F*, *t*, *r*) with confidence intervals, effect sizes, degrees of freedom and *P* value noted<br>*Give P values as exact values whenever suitable.*
☒	☐	For Bayesian analysis, information on the choice of priors and Markov chain Monte Carlo settings
☒	☐	For hierarchical and complex designs, identification of the appropriate level for tests and full reporting of outcomes
☒	☐	Estimates of effect sizes (e.g. Cohen's *d*, Pearson's *r*), indicating how they were calculated

Our web collection on statistics for biologists contains articles on many of the points above.

Software and code

Policy information about availability of computer code

Data collection	The cryo-EM data collections were performed at the University of Virginia Electron Microscopy Facility using EPU v2.8.
Data analysis	Initial rigid-body docking, model building and refinements were performed using UCSF ChimeraX v1.9, PHENIX v1.21.1-5419, and COOT v1.1.19. Model validation was performed using MolProbity v4.02b. All single-turnover kinetic experiments, electrophoretic mobility shift assays, and nucleosome stability assays were quantified and analyzed in ImageJ v1.54g. All MD simulations were performed and analyzed using the AMBER20 suite of programs. The code used to perform the flexibility analysis is available from https://github.com/emmanuellebignon/NCP-SIRT6/.

For manuscripts utilizing custom algorithms or software that are central to the research but not yet described in published literature, software must be made available to editors and reviewers. We strongly encourage code deposition in a community repository (e.g. GitHub). See the Nature Portfolio guidelines for submitting code & software for further information.

Data

Policy information about availability of data

All manuscripts must include a data availability statement. This statement should provide the following information, where applicable:

- Accession codes, unique identifiers, or web links for publicly available datasets
- A description of any restrictions on data availability
- For clinical datasets or third party data, please ensure that the statement adheres to our policy

Atomic coordinates for the reported structures have been deposited with the Protein Data Bank under accession numbers 10XZ, 10YA, 10YB, 10YC, 10YD, 10YE, 10YF, 10YG, 10YH, and 10YI. All cryo-EM maps are available from the Electron Microscopy Data Bank under accession numbers EMD-75521, EMD-75522, EMD-75523, EMD-75524, EMD-75525, EMD-75526, EMD-75527, EMD-75528, EMD-75529, EMD-75530, EMD-75531, EMD-75532, EMD-75533, EMD-75534, EMD-75535, EMD-75536, EMD-75537, and EMD-75538. Atomic coordinates for the initial nucleosome model and the LigIIIα-Nick-DNA model were obtained from the Protein Data bank under accession numbers 9DWF and 3L2P, respectively. The data from the single-turnover enzyme kinetics, EMSAs, and nucleosome stability assays generated in this study are available in the Source data file. The raw data from the MD simulations were deposited into the Zenodo repository and are available from https://doi.org/10.5281/zenodo.19235696. Source data are provided with this paper.

Research involving human participants, their data, or biological material

Policy information about studies with human participants or human data. See also policy information about sex, gender (identity/presentation), and sexual orientation and race, ethnicity and racism.

Reporting on sex and gender	This study did not involve research using human participants, their data, or biological material.
Reporting on race, ethnicity, or other socially relevant groupings	This study did not involve research using human participants, their data, or biological material.
Population characteristics	This study did not involve research using human participants, their data, or biological material.
Recruitment	This study did not involve research using human participants, their data, or biological material.
Ethics oversight	This study did not involve research using human participants, their data, or biological material.

Note that full information on the approval of the study protocol must also be provided in the manuscript.

Field-specific reporting

Please select the one below that is the best fit for your research. If you are not sure, read the appropriate sections before making your selection.

☒ Life sciences ☐ Behavioural & social sciences ☐ Ecological, evolutionary & environmental sciences

For a reference copy of the document with all sections, see nature.com/documents/nr-reporting-summary-flat.pdf

Life sciences study design

All studies must disclose on these points even when the disclosure is negative.

Sample size	Sample sizes were not predetermined based on statistical methods. Sample sizes for the single-turnover kinetics, electrophoretic mobility shift assays, nucleosome stability assays, and MD simulations were n = 3, which is based on the accepted standards within the field (see PMID: 28751376, PMID:17703195, PMID: 28202543, and PMID: 33057206). Sample size was not predetermined for the cryo-EM data collections. The sample size was determined by the number of particles that could be reasonably obtained during 1 or 2 days of data collection. The number of particles was sufficient to obtain cryo-EM reconstructions with overall resolutions of 3.5Å or better that were sufficient for model building and interpretation.
Data exclusions	Data exclusion was utilized on the cryo-EM datasets. Individual particles from the cryo-EM datasets were excluded during data processing and analysis. All cryo-EM datasets were subjected to extensive 2D and 3D classification during data processing to remove bad particles and/or bad classes of particles, which is standard within the cryo-EM community to enable generation of interpretable high-resolution cryo-EM maps (See PMID: 26705325). Data exclusion was not used for the biochemical assays or MD simulations.
Replication	The single-turnover kinetics, electrophoretic mobility shift assays, nucleosome stability assays, and MD simulations were performed as three independent replicate experiments to ensure reproducibility of the data. No data presented within this manuscript were unable to be reproduced.
Randomization	Randomization was not necessary for the experiments in this manuscript as no groups were allocated in our study. Therefore, controlling for covariates was not necessary.
Blinding	Blinding was not performed in these studies to ensure the correct samples were used for each respective experiment.

Reporting for specific materials, systems and methods

We require information from authors about some types of materials, experimental systems and methods used in many studies. Here, indicate whether each material, system or method listed is relevant to your study. If you are not sure if a list item applies to your research, read the appropriate section before selecting a response.

Materials & experimental systems

n/a	Involved in the study
☒	☐ Antibodies
☒	☐ Eukaryotic cell lines
☒	☐ Palaeontology and archaeology
☒	☐ Animals and other organisms
☒	☐ Clinical data
☒	☐ Dual use research of concern
☒	☐ Plants

Methods

n/a	Involved in the study
☒	☐ ChIP-seq
☒	☐ Flow cytometry
☒	☐ MRI-based neuroimaging

Plants

Seed stocks	This study did not involve research using plants.
Novel plant genotypes	This study did not involve research using plants.
Authentication	This study did not involve research using plants.
