# Supplementary material for: Molecular basis of nick ligation in the nucleosome by DNA Ligase IIIα
Source: Nat Commun. 2026 Sep 2;17:10460. doi: 10.1038/s41467-026-77207-7 (PMC13635136; doi:10.1038/s41467-026-77207-7)
Supplement: Supplementary file 3 [file 41467_2026_77207_MOESM3_ESM.pdf]

nature portfolio

# Peer Review File

# Molecular basis of nick ligation in the nucleosome by DNA Ligase IIIα

Corresponding Author: Dr Tyler Weaver

**This file contains all reviewer reports in order by version, followed by all author rebuttals in order by version.**

Version 0:

Reviewer comments:

Reviewer #1

(Remarks to the Author)
The manuscript is very well written, and the experiments are logical, clearly presented, and carefully executed. We have a few comments and suggestions for the authors:
• Figure 6 – complexes with XRCC1: No density for XRCC1 is observed in the cryo-EM structures, and the kinetics appear essentially identical to samples in the absence of XRCC1. Have the authors confirmed that a complex between XRCC1 and NCP–LigIIIα forms under their experimental conditions (e.g., via EMSA or analytical size-exclusion chromatography)?
• For the NCP nick structures in complex with LigIIIα, was focused 3D classification and/or refinement on the LigIIIα density attempted? The LigIIIα region appears to be at substantially lower resolution than the NCP in all cases; focused classification/refinement may improve this.
• Similarly, focused classification (as above) may help resolve the unassigned density reported in Supplementary Figure 18.
• Were efforts made to obtain reconstructions without the use of crosslinker? Given that LigIII has nanomolar affinity for nucleosome substrates, it seems plausible that the complex could be captured without crosslinking. If not, was adsorption to the air–water interface a limiting factor? A brief comment would be helpful.
• Please check abbreviation usage—terms introduced only once may not need to be abbreviated.
• Page 4: Typo – "active-site site chain" should read "active-site side chain."
• Line 104: Add "were used."
• Line 119: Clarify that specifically the NTase domain is bound, rather than the full-length protein.
• Line 199: Please provide a rationale for the choice of the 50 bp DNA substrate.
• Could AlphaFold prediction of full-length LigIIIα in complex with an NCP provide insight into the identity of the adjacent unassigned density? Even using an undamaged NCP model might yield informative predictions if confidence is sufficient.
• Line 424: Typo – extra "the."
• What does AlphaFold predict for the XRCC1–LigIIIα–NCP complex? Alternatively, if structural data exist for LigIIIα in complex with XRCC1, docking this into the reconstruction may reveal potential steric clashes. This could be analogous to previous observations where XRCC4 and LigIV domains clash with intact nucleosomes.
• As a potential additional experiment, the authors might consider expressing XRCC1 in insect cells to assess whether post-translational modifications influence its effect on ligation of nicked NCPs—particularly if prior literature suggests such an effect.

Reviewer #2

(Remarks to the Author)
In this manuscript, Boesch et al. explore how DNA ligase IIIα (LigIIIα) processes DNA nicks in the context of the nucleosome core particle (NCP), the basic unit of chromatin in which DNA is wrapped around a histone octamer. This is an important question for understanding how single-strand breaks, key intermediates in multiple DNA repair pathways, are repaired.

Overall, the study convincingly demonstrates that the NCP impedes DNA ligation. In the Widom DNA sequence context, subtle differences in ligation efficiency are observed depending on the precise location of solvent-exposed nicks within the NCP. For example, nick sites located near the NCP dyad are not ligated, whereas those near the entry/exit sites are ligated, but inefficiently compared to a nick in free DNA.

The study is rigorous and of high quality. Extensive controls, including affinity measurements of LigIIIα binding to nick-containing NCPs, five high-resolution cryoEM structures of intact and nicked NCPs, thermal stability assays, and one-microsecond atomistic MD simulations, support the conclusion that minimal changes in NCP stability and dynamics do not account for the observed differences in ligation kinetics.

The structural basis for nick recognition was determined from cryoEM structures of four nick-containing NCPs bound to LigIIIα, which reveal density mostly for the NTase domain, with some unassigned density. The structures show that, although LigIIIα can recognize nicks in all positions tested, the nucleosomal environment prevents full encirclement of the DNA by its multiple domains (DBD, NTase, and OBD), in contrast to what is seen with free DNA substrates.

A limitation of the study is that it does not provide a definitive explanation for how LigIIIα-mediated ligation occurs in chromatin, as the enzyme cannot fully encircle nucleosomal DNA. Because the Widom DNA sequence was used to assemble the NCPs, the authors could discuss whether its high positioning affinity may “overstabilize” the NCPs and thereby contribute (or not) to the inhibition of ligation. The authors propose several plausible hypotheses in the Discussion, including a particularly interesting possibility of “substrate channeling.” However, testing these ideas would require extensive additional work and is beyond the scope of the current manuscript, which already presents a lot of data. Despite this limitation, the study remains very valuable, as it rigorously demonstrates the inefficient ligation of solvent-exposed nicks within the NCP in vitro by LigIIIα or LigIIIα/XRCC1. This is an important step toward elucidating the complete ligation mechanism in chromatin. The work is technically sound, and the manuscript is clearly written.

Reviewer #3

(Remarks to the Author)
I co-reviewed this manuscript with one of the reviewers who provided the listed reports. This is part of the Nature Communications initiative to facilitate training in peer review and to provide appropriate recognition for Early Career Researchers who co-review manuscripts.

Reviewer #4

(Remarks to the Author)
In the work titled “Molecular basis of nick ligation in the nucleosome by DNA Ligase IIIα”, Boesch et al. use a combination of kinetic and binding studies, cryo-EM structures and molecular dynamics simulations to elucidate the molecular basis for nick ligation by LigIIIα in nucleosome core particles (NCP). The authors study four NCP complexes containing single strand breaks at four different superhelical locations (SHL0 ,SHL -2, SHL -4 and SHL-6) and find that the nicks that are located closest to entry/exit sites (Nick-NCP-6 and Nick-NCP-4) are ligated more robustly than nicks located closer to the dyad (Nick-NCP-2 and Nick-NCP0), despite no measurable differences in binding affinity of LigIIIα across these positions. Cryo-EM structures show that only the NTase domain of LigIIIα is well resolved, whereas the DBD and OBDs are disordered, indicating that LigIIIα is unable to adopt its catalytically competent conformation when bound to an NCP, suggesting a structural basis for the lower levels of ligation seen in NCPs compared to naked DNA.
The paper is well written, logically presented, and the data support the conclusions. This reviewer suggests publication after minor revisions.

(i) An introductory figure showing the steps described in lines 79-86 would be helpful to enhance understanding, perhaps include the role of the Mg2+in the activity of LigIIIα since this is pertinent for the structures reported later in the paper.

(ii) Line 90 - “nick ligation may represent the rate limiting step of chromatin-based SSBR/BER” is it not the ability of the BER enzyme to locate the damage? Perhaps nick ligation is the rate limiting step post damage recognition by the repair enzyme.

(iii) Figure 2b - please indicate whether the differences between the quantity of intact NCP remaining after the incubation of the different NCP-nick substrates are significant using p-values.

(iv) Figure 3- since the grey color indicates nucleosomal DNA according to the legend in panel d, perhaps the other domains of LigIIIα should be a different color.

(v) Line 228-229 LigIIIα only makes contact with the nucleosomal DNA and not the histone octamer. Is this because the positions chosen to introduce the nick are all outer positions. What would happen if the position was inner or mid?

(vi) This work uses the 601 sequence that is a tight binding sequence for histones. Is it possible that the tight binding of this DNA sequence to the histone octomer occludes access of LigIIIα to the nicked DNA. Would using natural centromeric sequences or perhaps chromatin remodelers improve ligation efficiency?

Reviewer #5

(Remarks to the Author)
I co-reviewed this manuscript with one of the reviewers who provided the listed reports. This is part of the Nature Communications initiative to facilitate training in peer review and to provide appropriate recognition for Early Career

Researchers who co-review manuscripts.

Version 1:

Reviewer comments:

Reviewer #1

(Remarks to the Author)
We are pleased that the authors have addressed all the points we raised. We are satisfied with the revisions and are happy to recommend the manuscript for publication.

Reviewer #2

(Remarks to the Author)
The authors have satisfactorily addressed all of the concerns raised, which were minor. This is a high-quality study, and we fully support its publication in its current form.

Reviewer #3

(Remarks to the Author)
I co-reviewed this manuscript with one of the reviewers who provided the listed reports. This is part of the Nature Communications initiative to facilitate training in peer review and to provide appropriate recognition for Early Career Researchers who co-review manuscripts.

Reviewer #4

(Remarks to the Author)
The authors have addressed my comments and concerns with updated figures and text. I believe this manuscript is suitable for publication at Nature Communications. Thank you.

Reviewer #5

(Remarks to the Author)
I co-reviewed this manuscript with one of the reviewers who provided the listed reports. This is part of the Nature Communications initiative to facilitate training in peer review and to provide appropriate recognition for Early Career Researchers who co-review manuscripts.

**Open Access** This Peer Review File is licensed under a Creative Commons Attribution 4.0 International License, which permits use, sharing, adaptation, distribution and reproduction in any medium or format, as long as you give appropriate credit to the original author(s) and the source, provide a link to the Creative Commons license, and indicate if changes were made.

In cases where reviewers are anonymous, credit should be given to 'Anonymous Referee' and the source.
The images or other third party material in this Peer Review File are included in the article’s Creative Commons license, unless indicated otherwise in a credit line to the material. If material is not included in the article’s Creative Commons license and your intended use is not permitted by statutory regulation or exceeds the permitted use, you will need to obtain permission directly from the copyright holder.
To view a copy of this license, visit https://creativecommons.org/licenses/by/4.0/

Reviewer #1 (Remarks to the Author):

**The manuscript is very well written, and the experiments are logical, clearly presented, and carefully executed. We have a few comments and suggestions for the authors:**

Response: We thank the reviewer for their enthusiasm and thorough evaluation of our work. We have addressed the comments/concerns of the reviewer in the point-by-point response below. All page and line numbers herein reference the location of the text within the revised manuscript. We have also highlighted regions of the revised manuscript with major textual changes in red.

1) **Figure 6 - complexes with XRCC1: No density for XRCC1 is observed in the cryo-EM structures, and the kinetics appear essentially identical to samples in the absence of XRCC1. Have the authors confirmed that a complex between XRCC1 and NCP-LigIIIα forms under their experimental conditions (e.g., via EMSA or analytical size-exclusion chromatography)?**

Response: We purified the XRCC1-LigIIIα complex by size-exclusion chromatography prior to performing the biochemical assays and cryo-EM. The XRCC1-LigIIIα complex coelutes together in a roughly 1:1 stoichiometry. We have added the chromatogram from the size exclusion chromatography and the corresponding SDS-PAGE gel highlighting the purity and stoichiometry of the complex (Supplementary Fig. 1e). Given the high affinity and stability of the XRCC1-LigIIIα complex (PMID: 8532526; PMID: 15987676), the inability to resolve XRCC1 in the cryo-EM reconstruction is likely due to conformational flexibility of XRCC1 relative to the LigIIIα-Nick-NCP complex rather than dissociation of the heterodimer. Consistently, we observed a shift in the migration of the XRCC1-LigIIIα-Nick-NCP complex compared to the LigIIIα-Nick-NCP during cryo-EM sample preparation (compare Supplementary Fig. 23a to Supplementary Fig. 5a), which strongly suggests that the XRCC1-LigIIIα heterodimer did not completely dissociate during complex formation.

**For the NCP nick structures in complex with LigIIIα, was focused 3D classification and/or refinement on the LigIIIα density attempted? The LigIIIα region appears to be at substantially lower resolution than the NCP in all cases; focused classification/refinement may improve this.**

Response: We appreciate the reviewer's suggestion and apologize for the lack of clarity regarding the use of focused 3D classification. Focused 3D classification and local refinement were both used for the LigIIIα-Nick-NCP-2, LigIIIα-Nick-NCP-4, LigIIIα-Nick-NCP-6, and XRCC1-LigIIIα-Nick-NCP-6 reconstructions. 3D classification was initially performed with a mask containing LigIIIα and the nucleosomal DNA surrounding the nick site to isolate classes with the highest LigIIIα occupancy. We then performed local refinement without particle subtraction using a mask encompassing the LigIIIα and the adjacent nucleosomal DNA. The resulting local refinement maps were then combined with the consensus LigIIIα-Nick-NCP maps using PHENIX combine focus maps to generate composite maps for model building and refinement. The resulting composite maps are the maps reported throughout the manuscript for the LigIIIα-Nick-NCP-2, LigIIIα-Nick-NCP-4, LigIIIα-Nick-NCP-6, and XRCC1-LigIIIα-Nick-NCP-6 complexes. Despite our best attempts, the local resolution of the LigIIIα within these reconstructions remains substantially lower than that of the nucleosome, which we attribute to a high level of conformational flexibility of LigIIIα and/or the nucleosomal DNA surrounding the nick site. This information can be found within the cryo-EM workflows (Supplementary Figs. 5-8 and 23) and we added text within the methods section (lines 602-610) to further clarify the use of focused 3D classification and local refinement with masks for LigIIIα and the adjacent nucleosomal DNA.

2) **Similarly, focused classification (as above) may help resolve the unassigned density reported in Supplementary Figure 18.**

Response: We appreciate this suggestion from the reviewer. We performed focused 3D classification and local refinement on the unassigned density adjacent to the LigIIIα NTase domain prior to submission of the manuscript. Unfortunately, the additional processing did not improve the maps in this region beyond what is presented in Supplementary Fig. 18.

3) **Were efforts made to obtain reconstructions without the use of crosslinker? Given that LigIII has nanomolar affinity for nucleosome substrates, it seems plausible that the complex could be captured without crosslinking. If not, was adsorption to the air-water interface a limiting factor? A brief comment would be helpful.**

Response: We did not make efforts to obtain reconstructions of the four LigIIIα-Nick-NCP complexes or the XRCC1-LigIIIα-Nick-NCP complex in the absence of crosslinking reagents for a variety of reasons. First, prior attempts to obtain structures of other upstream BER enzymes bound to their cognate lesions in the nucleosome (PMID: 36104361; PMID: 39715760; PMID: 39574623) without stabilization of the complexes using crosslinking reagents were unsuccessful. These complexes are purified via size exclusion chromatography prior to vitrification, and these enzymes dissociate from the damaged nucleosomes during the purification step despite the nanomolar affinities. This requirement for stabilizing BER/SSBR enzymes bound to damaged nucleosome to obtain cryo-EM reconstructions is also not unique to our lab, as other labs have also needed to stabilize these complexes for structure determination (PMID: 37339965; PMID: 39477986). Second, as previously noted by the reviewer, we observed substantial heterogeneity in LigIIIα and the nucleosomal DNA surrounding the nick site even in the complex stabilized by glutaraldehyde. We reasoned that collecting additional datasets in the absence of a crosslinking reagent would only enhance this heterogeneity, which would severely impact the interpretability of the cryo-EM reconstructions. Finally, collecting an additional 5 datasets in the absence of a crosslinking reagent would have required a substantial amount of additional time and resources, with no guarantee of additional structural insight. We do agree with the reviewer that this will be important to establish in the future, and we anticipate making further attempts to determine cryo-EM structures of LigIIIα bound to nicked nucleosomes, as well as other upstream base excision repair enzymes bound to their cognate lesions within the nucleosome in the absence of crosslinking reagents.

4) **Please check abbreviation usage—terms introduced only once may not need to be abbreviated.**

Response: We have reviewed the manuscript and confirm that all introduced abbreviations are used multiple times throughout the text.

5) **Page 4: Typo - “active-site site chain” should read “active-site side chain.”**

Response: This grammatical error has been fixed.

6) **Line 104: Add “were used.”**

Response: This grammatical error has been fixed.

**7) Line 119: Clarify that specifically the NTase domain is bound, rather than the full-length protein.**

Response: Unfortunately, line 119 does not seem to reference what the reviewer is pointing out. We think the reviewer is referring to the introductory paragraph describing the structures (lines 201-217), where we state that we determined cryo-EM reconstructions of LigIIIα bound to the four NCPs containing a nick at SHL-6, SHL-4, SHL-2, and SHL0. If this introductory paragraph is indeed what the reviewer is referencing, we completely understand the reviewer's point of view. We intentionally used LigIIIα (instead of LigIIIα NTase) to ensure the reader clearly understands that full-length LigIIIα was used during complex formation for cryo-EM structure determination. The following paragraph (lines 218-227) immediately clarifies that "*…of the three domains contributing to nick recognition (DBD, NTase, and OBD), we only observed clear density for the LigIIIα NTase domain in the reconstructions of LigIIIα bound to a nick in the nucleosome at SHL-6, SHL-4, SHL-2, and SHL0.*" We have chosen not to change this aspect of the manuscript as we are concerned this may lead to confusion for future readers of our work without the contextual information that is provided in the following paragraph.

**8) Line 199: Please provide a rationale for the choice of the 50 bp DNA substrate.**

Response: We appreciate this request for clarification regarding the use of the 50 bp Nick-DNA substrate. Prior single-turnover kinetics of nick ligation in DNA by LigIIIα were carried out with a 28 bp Nick-DNA substrate (PMID: 28751376). However, we were concerned that the 28 bp Nick-DNA substrate would not represent a great control for the 147 bp Nick-NCP substrates. We attempted to perform the control single-turnover ligation kinetics using a 147 bp Nick-DNA substrate. However, these longer oligonucleotides contained contaminants from synthesis (likely due to the length) that made interpreting the single turnover ligation kinetics quite challenging. Of note, these impurities do not impact the single turnover ligation kinetics in the nucleosome due to the additional purification steps that were taken after reconstituting the 147bp Nick-DNA into the nucleosome. We settled on performing the control single turnover ligation kinetics the 50 bp Nick-DNA as it still contains a substantial amount of additional DNA outside of the LigIIIα DNA binding footprint that would mimic the additional DNA within the nucleosome. Importantly, the ligation rate we obtained for the fast phase on the 50 bp Nick-DNA is essentially identical to the ligation rate previously determined for LigIIIα and a 28 bp Nick-DNA substrate under similar buffer conditions (PMID: 28751376), suggesting that DNA length likely has no impact or an extremely modest impact on LigIIIα ligation kinetics.

**9) Could AlphaFold prediction of full-length LigIIIα in complex with an NCP provide insight into the identity of the adjacent unassigned density? Even using an undamaged NCP model might yield informative predictions if confidence is sufficient.**

Response: We appreciate the reviewer's suggestion. However, it's unclear to us how performing AlphaFold modeling of LigIIIα with an undamaged nucleosome would inform on which LigIIIα domain the unassigned density in our reconstructions corresponds to, particularly given that the position of the unassigned density changes depending on nick location (see Supplementary Fig. 18). Given the close proximity of the unassigned density to the NTase domain in all reconstructions, and the fact that it appears to non-specifically interact with the nucleosomal DNA, we are fairly confident that the unassigned density belongs to either the DBD or OBD. Regardless, our reconstructions clearly show that neither domain is capable of encircling the DNA, which is critical for LigIIIα to adopt a catalytically active conformation. We strongly feel this conclusion stands irrespective of whether we can assign this additional density to a specific domain within LigIIIα.

It is worth pointing out that prior work did identify a putative interaction between the LigIIIα ZnF and the nucleosome acidic patch (PMID: 41271738), which was hypothesized to tether LigIIIα to the nucleosome while the enzyme attempts to engage the nucleosomal nick (see lines 402-406). However, we were unable to resolve an interaction between the LigIIIα ZnF and the nucleosome acidic patch in any of our cryo-EM reconstructions. The exact reason for this is unclear, but it's possible that LigIIIα cannot simultaneously engage the nick and acidic patch, or that the LigIIIα used in those experiments (nuclear extract from human cells) harbored LigIIIα post-translational modifications that promote the interaction of the ZnF with the nucleosome acidic patch. This is something we hope to explore further in the future.

**10) Line 424: Typo - extra "the."**

Response: This grammatical error has been fixed.

**11) What does AlphaFold predict for the XRCC1-LigIIIα-NCP complex? Alternatively, if structural data exist for LigIIIα in complex with XRCC1, docking this into the reconstruction may reveal potential steric clashes. This could be analogous to previous observations where XRCC4 and LigIV domains clash with intact nucleosomes.**

Response: Substantial biochemical and structural studies have established that XRCC1 interacts with LigIIIα exclusively through a BRCT-BRCT domain interface (see PMID: 9705932; PMID: 21652643; PMID: 33330937). Importantly, the BRCT domain of LigIIIα is connected to the catalytic core by a relatively long and flexible linker. Given the nature of the known XRCC1-LigIIIα dimerization interface, we reasoned that visualizing XRCC1 within the complex would likely require that XRCC1 directly interact with the nucleosome itself, which is clearly not the case under our experimental conditions. However, it's possible the interaction of XRCC1 with the nucleosome may be facilitated by the presence of histone ADP-ribosylation, as XRCC1 was previously shown to be recruited to chromatin via a direct interaction with poly-ADP-ribose chains (see PMID: 14500814; PMID: 27965414; PMID: 30650352). The interplay between the XRCC1-LigIIIα complex, histone ADP-ribosylation, and nick ligation was a point we discussed in the manuscript (see lines 396-399), and is something we hope to further address in the future.

**12) As a potential additional experiment, the authors might consider expressing XRCC1 in insect cells to assess whether post-translational modifications influence its effect on ligation of nicked NCPs—particularly if prior literature suggests such an effect.**

Response: We appreciate the reviewer's suggestion, and we agree that it would be informative to test whether insect cell-derived XRCC1 (with post-translational modifications) behaves differently from the bacterially expressed XRCC1 used in our study, particularly given the subtle stimulation in ligation efficiency observed previously (PMID: 21930793; PMID: 28184006). However, producing insect cell-derived XRCC1 and performing the biochemical assays and cryo-EM would require a substantial amount of additional time and resources that we strongly feel are outside the scope of the manuscript.

Reviewer #2 (Remarks to the Author):

**In this manuscript, Boesch et al. explore how DNA ligase IIIα (LigIIIα) processes DNA nicks in the context of the nucleosome core particle (NCP), the basic unit of chromatin in which DNA is wrapped around a histone octamer. This is an important question for understanding how single-strand breaks, key intermediates in multiple DNA repair pathways, are repaired.**

**Overall, the study convincingly demonstrates that the NCP impedes DNA ligation. In the Widom DNA sequence context, subtle differences in ligation efficiency are observed depending on the precise location of solvent-exposed nicks within the NCP. For example, nick sites located near the NCP dyad are not ligated, whereas those near the entry/exit sites are ligated, but inefficiently compared to a nick in free DNA.**

**The study is rigorous and of high quality. Extensive controls, including affinity measurements of LigIIIα binding to nick-containing NCPs, five high-resolution cryoEM structures of intact and nicked NCPs, thermal stability assays, and one-microsecond atomistic MD simulations, support the conclusion that minimal changes in NCP stability and dynamics do not account for the observed differences in ligation kinetics.**

**The structural basis for nick recognition was determined from cryoEM structures of four nick-containing NCPs bound to LigIIIα, which reveal density mostly for the NTase domain, with some unassigned density. The structures show that, although LigIIIα can recognize nicks in all positions tested, the nucleosomal environment prevents full encirclement of the DNA by its multiple domains (DBD, NTase, and OBD), in contrast to what is seen with free DNA substrates.**

**A limitation of the study is that it does not provide a definitive explanation for how LigIIIα-mediated ligation occurs in chromatin, as the enzyme cannot fully encircle nucleosomal DNA. Because the Widom DNA sequence was used to assemble the NCPs, the authors could discuss whether its high positioning affinity may "overstabilize" the NCPs and thereby contribute (or not) to the inhibition of ligation. The authors propose several plausible hypotheses in the Discussion, including a particularly interesting possibility of "substrate channeling." However, testing these ideas would require extensive additional work and is beyond the scope of the current manuscript, which already presents a lot of data. Despite this limitation, the study remains very valuable, as it rigorously demonstrates the inefficient ligation of solvent-exposed nicks within the NCP in vitro by LigIIIα or LigIIIα/XRCC1. This is an important step toward elucidating the complete ligation mechanism in chromatin. The work is technically sound, and the manuscript is clearly written.**

Response: We thank the reviewer for their thorough and thoughtful evaluation of our manuscript and for recognizing the rigor and quality of our work. We are pleased that the reviewer found the study valuable and the conclusions well-supported by the data. We also appreciate the reviewer's understanding that further experiments to directly observe LigIIIα-mediated ligation in the nucleosome are beyond the scope of this manuscript, though we do anticipate continuing to work on this in the future.

We also agree with the reviewer that the use of the Widom 601 strong positioning sequence could overestimate the extent of LigIIIα nick ligation inhibition within the nucleosome, particularly at positions in proximity to the nucleosome dyad where we observed minimal LigIIIα activity (i.e.,

SHL$_{-2}$ and SHL$_0$). This is an important point that was also brought up by Reviewer #4. To address this, we generated NCPs containing a nick at SHL$_{-2}$ and SHL$_0$ using the 5S rDNA sequence from *Xenopus borealis*. We then performed single-turnover kinetics for LigIIIα on the *5S*-Nick-NCP$_{-2}$ and *5S*-Nick-NCP$_0$ substrates. Of note, we observed minimal LigIIIα nick ligation (<10%) on the *5S*-Nick-NCP$_{-2}$ and *5S*-Nick-NCP$_0$ substrates, consistent with what we observed for LigIIIα on the 601 strong positioning sequence. This new data strongly suggests that the inability of LigIIIα to perform nick ligation at translational positions outside of the nucleosome entry/exit site is not a consequence of the 601 strong positioning sequence. This new data has been added to the manuscript (see lines 134-138, Supplementary Fig. 1c, and Supplementary Fig. 2c,d, and Supplementary Table 1).

Reviewer #3 (Remarks to the Author):

**I co-reviewed this manuscript with one of the reviewers who provided the listed reports. This is part of the Nature Communications initiative to facilitate training in peer review and to provide appropriate recognition for Early Career Researchers who co-review manuscripts.**

Response: We thank the Early Career Reviewer for the time and effort spent reviewing our manuscript.

Reviewer #4 (Remarks to the Author):

**In the work titled "Molecular basis of nick ligation in the nucleosome by DNA Ligase IIIα", Boesch et al. use a combination of kinetic and binding studies, cryo-EM structures and molecular dynamics simulations to elucidate the molecular basis for nick ligation by LigIIIα in nucleosome core particles (NCP). The authors study four NCP complexes containing single strand breaks at four different superhelical locations (SHL0 ,SHL -2, SHL -4 and SHL-6) and find that the nicks that are located closest to entry/exit sites (Nick-NCP-6 and Nick-NCP-4) are ligated more robustly than nicks located closer to the dyad (Nick-NCP-2 and Nick-NCP0), despite no measurable differences in binding affinity of LigIIIα across these positions. Cryo-EM structures show that only the NTase domain of LigIIIα is well resolved, whereas the DBD and OBDs are disordered, indicating that LigIIIα is unable to adopt its catalytically competent conformation when bound to an NCP, suggesting a structural basis for the lower levels of ligation seen in NCPs compared to naked DNA.**

**The paper is well written, logically presented, and the data support the conclusions. This reviewer suggests publication after minor revisions.**

Response: We thank the reviewer for their overall enthusiasm for our work. We have addressed the comments/concerns of the reviewer in the point-by-point response below. All page and line numbers herein reference the location of the text within the revised manuscript. We have also highlighted regions of the revised manuscript with major textual changes in red.

1. **An introductory figure showing the steps described in lines 79-86 would be helpful to enhance understanding, perhaps include the role of the Mg2+in the activity of LigIIIα since this is pertinent for the structures reported later in the paper.**

Response: We agree with the reviewer that this would be helpful for future readers of our manuscript. Figure 1a is now a cartoon diagram of the established ligation mechanism described in lines 76 to 85.

2. **Line 90 - "nick ligation may represent the rate limiting step of chromatin-based SSBR/BER" is it not the ability of the BER enzyme to locate the damage? Perhaps nick ligation is the rate limiting step post damage recognition by the repair enzyme.**

Response: We thank the reviewer for pointing out the lack of clarity in this statement. This statement was meant to describe the idea that LigIIIα nick ligation is inhibited in the nucleosome to a greater extent than upstream SSBR/BER. We have revised the text to clarify that nick ligation likely represents the rate-limiting enzymatic step of chromatin-based SSBR/BER (lines 89 and 354).

3. **Figure 2b - please indicate whether the differences between the quantity of intact NCP remaining after the incubation of the different NCP-nick substrates are significant using p-values.**

Response: We thank the reviewer for pointing out this issue. We have performed the statistical analysis comparing the amount of intact Nick-NCP to the amount of intact ND-NCP after the 24-hour incubation using a Student's T-test. The statistical analysis indicates that there is not a

significant difference in the amount of intact Nick-NCP compared to intact ND-NCP after the 24-hour incubation. Fig 2b and the associated figure legends have been adjusted accordingly.

4. **Figure 3- since the grey color indicates nucleosomal DNA according to the legend in panel d, perhaps the other domains of LigIIIα should be a different color.**

Response: We appreciate the reviewer's suggestion. We have changed the color scheme of the domain architecture diagram in Fig. 3a and Fig. 6a so that the unresolved LigIIIα domains are colored in pale orange. We have also updated the figure legend to clarify that the LigIIIα domains colored in pale orange represent domains that were not resolved in the cryo-EM reconstructions (lines 1059-1061 and 1105-1107).

5. **Line 228-229 LigIIIα only makes contact with the nucleosomal DNA and not the histone octamer. Is this because the positions chosen to introduce the nick are all outer positions. What would happen if the position was inner or mid?**

Response: We thank the reviewer for this interesting question. We believe that the absence of histone contacts is not a consequence of the rotational positions chosen but rather reflects the intrinsic mode of nucleosomal DNA recognition by the LigIIIα. It's not completely clear how common histone-occluded nicks would be given that direct SSBs stemming from oxidation-induced disintegration of the sugar phosphate backbone have a strong preference for forming at solvent exposed positions in the nucleosome (PMID: 2170977; PMID: 37527775). In addition, SSBs arising as BER intermediates preferentially form at solvent-exposed positions due to the relatively poor activity of upstream BER enzymes on histone occluded lesions in the nucleosome (reviewed extensively in PMID: 37527775; PMID: 41161019). However, if LigIIIα were to encounter a fully or partially histone-occluded nick, we envision LigIIIα would be unable to engage the nick without substantial alterations in the nucleosomal DNA to prevent clashing between the LigIIIα NTase domain and the histone octamer.

We would like to point out that prior work did identify a putative interaction between the LigIIIα ZnF and the nucleosome acidic patch (PMID: 41271738), which was hypothesized to tether LigIIIα to the nucleosome during nick recognition. However, we were unable to resolve an interaction between the LigIIIα ZnF and the nucleosome acidic patch in any of our cryo-EM reconstructions. The exact reason for our inability to resolve this interaction is unclear, but something we hope to explore further in the future.

6. **This work uses the 601 sequence that is a tight binding sequence for histones. Is it possible that the tight binding of this DNA sequence to the histone octomer occludes access of LigIIIα to the nicked DNA. Would using natural centromeric sequences or perhaps chromatin remodelers improve ligation efficiency?**

Response: The reviewer brings up an excellent point, which was also brought up by Reviewer #2. It's reasonable to suggest the use of Widom 601 strong positioning sequence could overestimate the extent of LigIIIα nick ligation inhibition within the nucleosome, particularly at positions in proximity to the nucleosome dyad where we observed minimal activity (i.e., $SHL_{-2}$ and $SHL_0$). To address this, we generated NCPs containing a nick at $SHL_{-2}$ and $SHL_0$ using the 5S rDNA sequence from *Xenopus borealis* (see Supplementary Fig. 1c). We then performed additional single-turnover kinetics experiments for LigIIIα and the *5S*-Nick-$NCP_{-2}$ and *5S*-Nick-$NCP_0$ substrates (see Supplementary Fig. 2c,d, and Supplementary Table 1). Of note, we observed minimal LigIIIα nick ligation (<10%) on the *5S*-Nick-$NCP_{-2}$ and *5S*-Nick-$NCP_0$ substrates, consistent with what we observed for LigIIIα on the 601 strong positioning sequence. This new

data strongly suggests that the inability of LigIIIα to perform nick ligation at translational positions outside of the nucleosome entry/exit site is not a consequence of the 601 strong positioning sequence. This new data has been added to the manuscript (see lines 134-138, Supplementary Fig. 1c, Supplementary Fig. 2c,d, and Supplementary Table 1).

We completely agree with the reviewer that chromatin modifying enzymes will likely have a profound impact on the ability of LigIIIα to function within the nucleosome. We dedicated a substantial portion of the discussion to this exact idea (see lines 393-402). We anticipate further exploring the complex interplay between DNA excision repair enzymes and chromatin modifying enzymes in the future.

Reviewer #5 (Remarks to the Author):

**I co-reviewed this manuscript with one of the reviewers who provided the listed reports. This is part of the Nature Communications initiative to facilitate training in peer review and to provide appropriate recognition for Early Career Researchers who co-review manuscripts.**

Response: We thank the Early Career Reviewer for the time and effort spent reviewing our manuscript.

Reviewer #1 (Remarks to the Author):

**We are pleased that the authors have addressed all the points we raised. We are satisfied with the revisions and are happy to recommend the manuscript for publication.**

Response: We thank the Reviewer for the time and effort spent reviewing our manuscript.

Reviewer #2 (Remarks to the Author):

**The authors have satisfactorily addressed all of the concerns raised, which were minor. This is a high-quality study, and we fully support its publication in its current form.**

Response: We thank the Reviewer for the time and effort spent reviewing our manuscript.

Reviewer #3 (Remarks to the Author):

**I co-reviewed this manuscript with one of the reviewers who provided the listed reports. This is part of the Nature Communications initiative to facilitate training in peer review and to provide appropriate recognition for Early Career Researchers who co-review manuscripts.**

Response: We thank the Early Career Reviewer for the time and effort spent reviewing our manuscript.

Reviewer #4 (Remarks to the Author):

**The authors have addressed my comments and concerns with updated figures and text. I believe this manuscript is suitable for publication at Nature Communications. Thank you.**

Response: We thank the Reviewer for the time and effort spent reviewing our manuscript.

Reviewer #5 (Remarks to the Author):

**I co-reviewed this manuscript with one of the reviewers who provided the listed reports. This is part of the Nature Communications initiative to facilitate training in peer review and to provide appropriate recognition for Early Career Researchers who co-review manuscripts.**

Response: We thank the Early Career Reviewer for the time and effort spent reviewing our manuscript.
